# Interplay of Rotational and Pseudorotational Motions in Flexible Cyclic Molecules

**DOI:** 10.1021/acs.jpca.1c01472

**Published:** 2021-05-11

**Authors:** Lorenzo Paoloni, Assimo Maris

**Affiliations:** † Dipartimento di Fisica e Astronomia, Università di Padova, via Marzolo 8, I-35131 Padova, Italy; ‡ Dipartimento di Chimica G. Ciamician, Università di Bologna, via Selmi 2, I-40126 Bologna, Italy

## Abstract

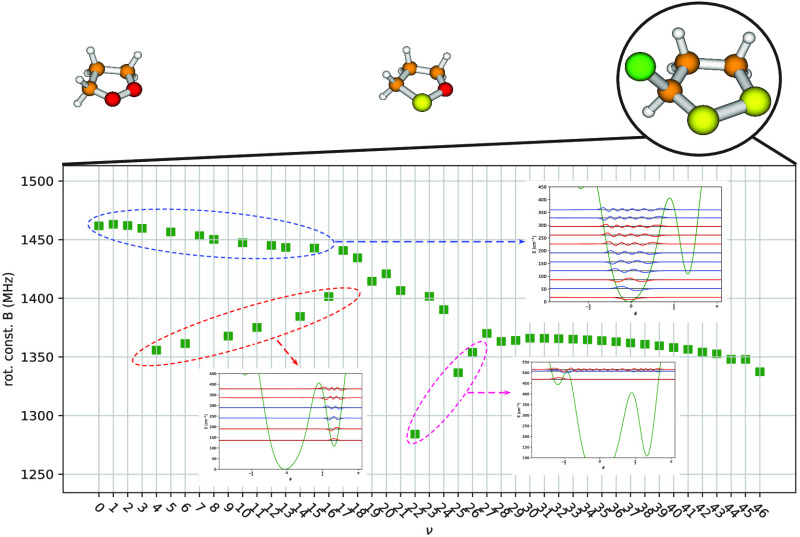

Solutions to the time-independent nuclear Schrödinger equation associated with the pseudorotational motion of three flexible cyclic molecules are presented and discussed. Structural relaxations related to the pseudorotational motion are described as functions of a pseudorotation angle ϕ which is formulated according to the definition of ring-puckering coordinates originally proposed by Cremer and Pople (J. Am. Chem. Soc.1975, 97 ( (6), ), 1354−1358). In order to take into account the interplay between pseudorotational and rotational motions, the rovibrational Hamiltonian matrices are formulated for the rotational quantum numbers *J* = 0 and *J* = 1. The rovibrational Hamiltonian matrices are constructed and diagonalized using a Python program developed by the authors. Suitable algorithms for (i) the construction of one-dimensional cuts of potential energy surfaces along the pseudorotation angle ϕ and (ii) the assignment of the vibrorotational wave functions (which are needed for the automatic calculation of rotational transition energies *J* = 0 → *J* = 1) are described and discussed.

## 1 Introduction

The relevance of intramolecular nuclear motion effects on the physical chemical properties of isolated cyclic molecules is well-known.^1−3^ A confirmation of the importance of this phenomenon is provided by the analysis of the experimental spectra of cyclic molecular systems. Some of these experimental data were rationalized as consequences of ring puckering (RP) motions (for example, see refs (4−8)), which in general are not easily described in terms of Cartesian or primitive internal coordinates (i.e., in terms of bond lengths or dihedral/valence angle variations) of nuclei. Many efforts^9−12^ were devoted to the formulation of a curvilinear coordinate system suitable for a general description of RP motions in cyclic molecular systems.

Nowadays, RP motions of *N*-membered rings (with *N* equal to or greater than 5) can be elegantly described with Cremer–Pople coordinates (CPCs).^12^ With the definitions proposed by Cremer and Pople, the RP of a cyclic moiety comprising *N* atoms (the substituents are not taken into account in this number, i.e. *N* = 5 atoms pertains to the cyclic moiety of cyclopentane) can be described with *N* – 3 CPCs. After the introduction of ring deformation coordinates (RDCs) by Zou, Izotov, and Cremer,^13^ the conformation of a cyclic moiety with *N* atoms can be completely specified employing *N* – 3 CPCs and 2*N* – 3 RDCs.

In the special case of a 5-term ring system, RP can be described with 2 CPCs and visualized with a two-dimensional cut of the global potential energy surface (2D-PES).^14^ An analysis of the RP motions based on the separation of the RP motions from the other vibrational motions of the molecular system of interest is an approximation. Nevertheless, this approximation is often reliable and useful. In the framework of the Born–Oppenheimer (BO) approximation, the reduction in dimensionality achieved for the PES of a 5-term ring system by employing CPCs can be extended to the solution of the time-independent nuclear Schrödinger equation (NSE). In other words, the 2D-PES which describes the RP of a 5-term ring system can be employed in the formulation of the NSE associated with the RP of the same system. The picture can be further simplified: as observed in previous works,^15^ the minimum-energy path associated with RP motions of 5-term saturated ring systems can be often described with a single CPC, namely, the pseudorotation angle ϕ.

**Figure 1:**
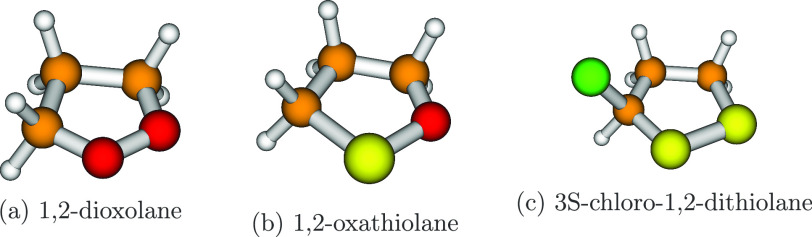
Planar structures of the three molecular systems studied in this work.

In this work, the solutions of the NSE associated with the Cremer–Pople pseudorotation angle ϕ for the three molecular systems shown in Figure 1 are presented and discussed. First, an algorithm suitable for the construction of a one-dimensional cut of the global PES (1D-PES) along the pseudorotation angle ϕ was devised, implemented, and employed (in order to derive the potential energy term of the nuclear Hamiltonian). Second, the eigenvalue problem related to the nuclear Hamiltonian was solved with a computational strategy based on the employment of a monodimensional discrete variable representation (DVR).^16^ More specifically, the strategy originally proposed by Meyer in refs (17 and 18) was employed.

The solutions of the nuclear problem related to the pseudorotational pathway are provided for two values of the rotational quantum number *J*; that is, the nuclear Hamiltonian was constructed for *J* = 0 (pure psuedorotational case) and *J* = 1. In this way, the interplay between pseudorotational and rotational motions can be numerically evaluated and discussed.

The model employed in this work to reduce the full dimensional NSE to a simpler 1D problem is named *flexible model* by Meyer^19^ and *semirigid model*(20) or *adiabatic-constraint approach*(21,22) by other authors. There are other approaches to dimensionality reduction of the nuclear problem (a list of useful references and a brief historical account concerning development and employment of approximated separation methods for the description of molecular motions can be found in the introduction of chapter 12 of ref (23)). In particular, the employment of a BO-like separation between small amplitude motions (SAMs) and large amplitude motions (LAMs) was proposed:^24−26^ in this approach, the formulation of an effective nuclear Hamiltonian for the LAM of interest is used to take into account the effects of SAMs (completely neglected in this work) in the solutions of the nuclear problem. Furthermore, higher-dimensional formulation of the nuclear problem can be achieved: in the original article by Meyer,^19^ an extension to the 2D problem was proposed, and other extensions suitable for the solution of more complex formulations of the nuclear problems (*J* > 1 and more than two coupled internal coordinates) are available in literature.^27−29^ Nowadays, even the solution of the full dimensional problem in curvilinear coordinates is possible,^30^ at least in principle (in practice, to formulate the full dimensional problem the calculation of the global PES must be feasible). An up-to-date picture of the currently available nonperturbative approaches to the formulation of a nuclear Hamiltonian and to the solution of the final eigenvalue problem can be found in ref (31).

This article is structured as follows. In section 2, the definition of CPCs is introduced and the method employed for the solution of the NSE associated with the pseudorotation angle ϕ is discussed. In section 3, the computational protocol is outlined. In section 4, computational results are discussed: eigenvalues and eigenvectors of the nuclear Hamiltonians are provided and commented on for each of the compounds shown in Figure 1. In section 5, accuracy, limitations, and possible improvements of the model are discussed. Finally, conclusions are outlined in section 6.

## 2 Theoretical Background

As mentioned in the Introduction, RP motions of flexible cyclic molecular systems can be described with CPCs. The definition of CPCs was proposed for the first time in ref (12). In what follows, the definition of CPCs is provided for the special case of a 5-term ring system. In this special case, only 2 CPCs are required: *q* (the puckering amplitude) and ϕ (the pseudorotation angle). To define *q* and ϕ, the Cartesian framework employed to specify the nuclear positions of the molecular system must be translated and rotated. More specifically, the origin of the Cartesian framework must coincide with the geometrical center of the 5 nuclei directly involved in the ring structure:^12^
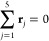
1where **r**_*j*_ specifies the position of the *j*th atomic nucleus; then, the *z* axis must be oriented perpendicularly to a mean plane. The position vectors **r***′* and **r***″* can be employed to fix the orientation of the mean plane and the orientation of the **ẑ** versor, which is perpendicular to the mean plane:^12^
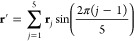
2
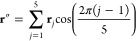
3
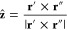
4In a Cartesian framework translated and oriented according to eqs 1–4, the coordinate *z*_*j*_ of the *j*th atomic nucleus is defined as follows:
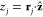
5The length *q* and the pseudorotation angle ϕ are defined in the following manner:^12,32,33^
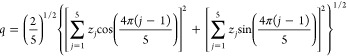
6
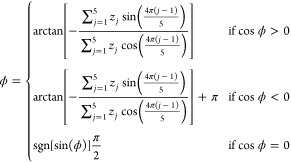
7

**Figure 2:**
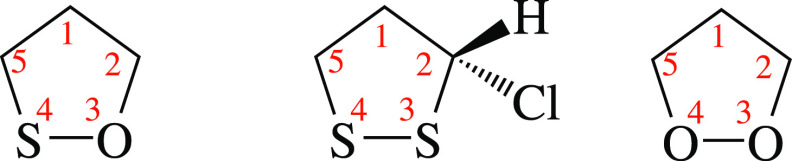
Sketch and numbering of the atoms involved in the definition of CPCs in 1,2-oxathiolane, 3S-chloro-1,2-dithiolane, and 1,2-dioxolane.

In this work, the conformation of a 5-term ring system is specified through the CPCs *q* and ϕ. In order to establish a univocal correspondence between the value of the pseudorotation angle ϕ and the conformation of a 5-term ring system, the sequential numbering of the 5 atoms directly involved in the ring structure must be specified (see Figure 2). In other words, each atom is labeled with a specific *j* value (from 1 to 5), and two consecutive *j* values must be assigned to adjacent atoms of the ring.

**Figure 3:**
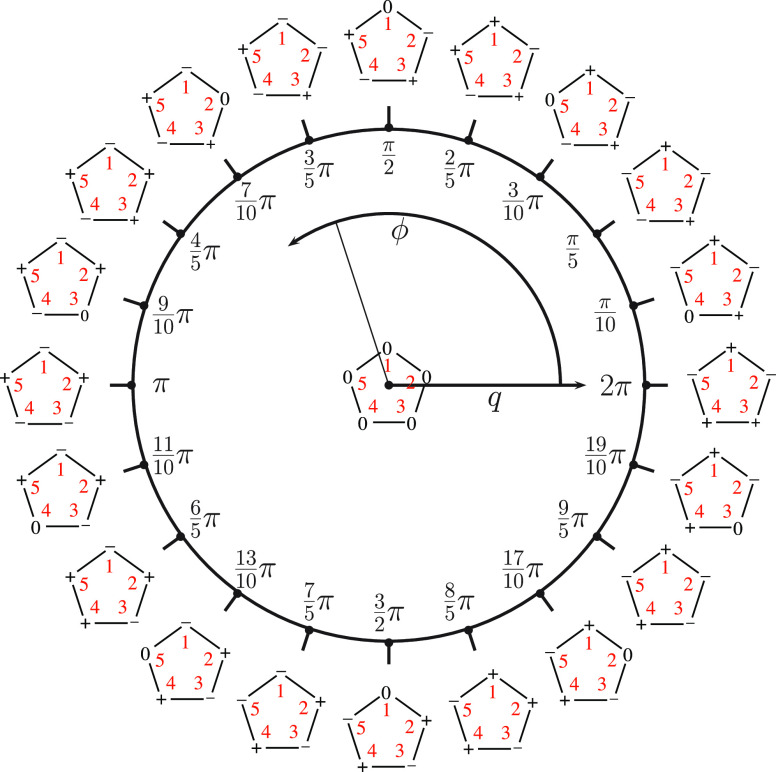
Conformations of a 5-term ring system at different values of the pseudorotation angle ϕ.

A graphical representation of the correspondence between a couple of values (*q*, ϕ) and the conformation of a 5-term ring system can be found in Figure 3. The planar conformation (*q* = 0) corresponds to the center of the circle.

Other coordinate systems were employed by other authors to explore 2D-PES of 5-term ring systems.^34,35^ For example, the same conformational space spanned by *q* and ϕ can be explored defining another two coordinates, *q* cos ϕ and *q* sin ϕ (sometimes called ring-twisting and ring-bending coordinates). However, in many cases the minimum-energy path can be described as a one-dimensional pseudorotation pathway: in these cases, a well-defined pseudorotation angle ϕ can be usefully employed to construct a 1D-PES. This simple idea was recognized before the pivotal article of Cremer and Pople^12^ (for example, see ref (9)), but the general definition of the pseudorotation angle proposed in ref (12) is the starting point for a systematic exploration of pseudorotational pathways in 5-term ring systems. Nowadays, the availability of analytic gradients of CPCs^32^ allows the construction of a 1D-PES along the pseudorotation angle ϕ defined in eq 7 (for example, see ref (36)). In this article, constrained geometry optimizations were performed at different values of ϕ. The constraints were automatically generated with the algorithm proposed in section 1.1 of the Supporting Information: during each optimization, the ϕ value was fixed while all the other nuclear degrees of freedom (including *q*) were optimized. In this manner, the points of a 1D-PES were calculated.

As mentioned above, dimensionality reduction allows for a formulation of the potential energy term *V̂* in terms of a limited number of coordinates (typically one or two, i.e., construction of 1D-PES or 2D-PES). This is the starting point for the construction of a nuclear Hamiltonian *Ĥ* employed to describe nuclear motions in terms of a reduced coordinate space.^37−39^ This is an approximated approach: in general, a molecular system is subject to many nuclear motions which cannot be described with a reduced set of one or two curvilinear nuclear coordinates. However, the coupling is often negligible, and a separation between LAMs and SAMs can be introduced. In this work, the pseudorotation of a 5-term ring system is described as a LAM, and its separability from the other internal nuclear degrees of freedom is assumed. In other words: if the number of nuclei in the molecular system is equal to *K*, the coupling between the pseudorotational motion and the other 3*K* – 7 internal degrees of freedom is neglected. The problem is therefore reduced to the solution of a one-dimensional NSE (1D-NSE) associated with the pseudorotational motion:
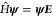
8

The nuclear Hamiltonian *Ĥ* can be partitioned as follows:
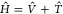
9where *V̂* is the potential energy term and *T̂* the kinetic energy operator (KEO). The employment of a curvilinear coordinate system is often advantageous for the exploration of the conformational space of a molecular system (i.e., for the construction of *V̂*). On the other hand, the analytical expression of the KEO in curvilinear coordinates^40−42^ can be extremely complicated. In other words, while a simple analytical formulation of the KEO in normal coordinates is well-known,^43−45^ attempts to derive an analytical expression for the KEO in curvilinear coordinates led to complex and lengthy formulations.^46−48^ Some authors proposed an alternative approach,^49^ which was adopted also for this work: the numerical computation of the KEO in curvilinear coordinates. Another possibility is the employment of computer procedures based on symbolic calculations in order to achieve an automatic derivation of an analytical formulation of the KEO.^50,51^

The pragmatic approach chosen for this work was proposed and developed by Meyer.^17−19^ In particular, the computational strategy proposed in ref (19) was adopted. The construction of the nuclear Hamiltonian matrices is detailed in section 2 of the Supporting Information. In order to take into account the interaction between pseudorotational and rotational motions, two formulations of the nuclear Hamiltonian were employed. The first formulation is suitable for the solution of a pure vibrational problem (i.e., the 1D-NSE related to the pure pseudorotational motion), which is the case of *J* = 0:
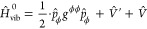
10where *V̂*^′^ is a pseudopotential term (originally introduced in ref (52)) which allows for a more compact formulation of the KEO, , and *g*^*ϕϕ*^ is an element of the kinetic energy matrix (which is introduced in section 2 of the Supporting Information, see also eq 3.4 of ref (19)). The second formulation is employed for the solution of the vibro-rotational problem in the case *J* = 1:
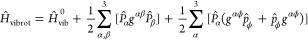
11where *g*^*αβ*^ and *g*^*αϕ*^ are elements of the kinetic energy matrix and *P̂*_α_ is a component of the overall angular momentum with respect to molecule-fixed axis.

The 1D-NSE can be viewed as an eigenvalue problem: the eigenvectors are the wave functions (**ψ**) and the eigenvalues are the energies (***E***).

## 3 Computational Protocol

Electronic calculations and constrained geometry optimizations were performed using the Gaussian 16 suite of programs.^53^ Electronic calculations were carried out with density functional theory (DFT) by employing B3LYP^54−56^ as an exchange–correlation functional and maug-cc-pVTZ basis set.^57,58^ Each point of the 1D-PES was calculated independently through a constrained optimization. As in ref (33), input geometries were constructed using both Cartesian and primitive internal coordinates (PICs, i.e., bond lengths and dihedral and valence angles) in the same Z-matrix. The positions of the 5 atoms directly involved in the ring structure (e.g., carbon and oxygen atoms in the case of 1,2-dioxolane) were specified with Cartesian coordinates (translated and oriented according to eqs 1–4), while the positions of the other atoms were provided in terms of PICs (e.g., hydrogen atoms in the case of 1,2-dioxolane). In the case of ref (33), both *q* and ϕ were fixed during the constrained optimization (i.e., 2D-PESs were constructed). In this work, only ϕ is fixed, while *q* is optimized: therefore, the homemade Python script mentioned in section 3 of ref (33) and employed to automatically write input files corresponding to a specific couple (*q*, ϕ) was modified accordingly. In practice, the algorithm discussed in section 1.1 of the Supporting Information was devised and implemented. In contrast to the protocol of ref (33) (*z*_*j*_ Cartesian coordinates of the 5 atoms directly involved in the ring structure are kept fixed), the following 4 constraints are imposed during the geometry optimization: three ratios *z*_*j*_/*z*_*j*′_ involving 4 of the 5 *z*_*j*_ Cartesian coordinates of the atoms directly involved in the ring structure (the *z*_*j*_ coordinate with the lower absolute value is excluded) are kept fixed, and the sum of the 5 *z*_*j*_ Cartesian coordinates is constrained to 0, according to eq 1. For more details, see section 1.1 of the Supporting Information.

For each of the molecular systems investigated in this work, 360 constrained geometry optimizations were carried out (each one corresponding to a specific value of the pseudorotation angle ϕ, which is kept fixed during the optimization, i.e., for ϕ equal to 1°, 2°, ..., 360°) with extremely tight convergence criteria (RMS values of 1 × 10^–6^ hartree/bohr and 4 × 10^–6^ bohr and maximum values of 2 × 10^–6^ hartree/bohr and 6 × 10^–6^ bohr on forces and displacements, respectively). Molecular structures and energies obtained with the constrained geometry optimizations were extracted and employed for the construction of the nuclear Hamiltonian matrices for the cases *J* = 0 (*Ĥ*_vib_^0^) and *J* = 1 (*Ĥ*_vibrot_^1^). Data extraction from the Gaussian outputs, construction of the nuclear Hamiltonian matrices (a formulation suitable for an implementation of the matrix elements  and  is provided in eqs SI-42 and SI-79, respectively; see the Supporting Information), solution of the eigenvalue problems, and data analysis were performed with a Python program written by the authors. In order to improve the computational efficiency of the code, Fortran90 subroutines were implemented for the numerical calculation of the nuclear Hamiltonian matrix *Ĥ*_vibrot_^1^. F2PY^59^ was employed to interface Fortran90 subroutines to the main Python program.

With regard to the solution of the one-dimensional nuclear problem (eqs 8 and 9), the principal axis system (PAS) was adopted as the reference configuration of the Cartesian framework for each optimized molecular structure.

## 4 Computational Results

In this section, solutions of the 1D-NSE related to the pseudorotational angle ϕ are provided and discussed. More specifically, the solutions of the eigenvalue problems associated with the nuclear Hamiltonian matrices *Ĥ*_vib_^0^ (for the case *J* = 0) are provided for each of the three molecular systems shown in Figure 1, together with rotational constants and rotational transition energies (for the case *J* = 0 → *J* = 1). A previous computational study concerning 1,2-dioxolane, 1,2-oxathiolane, and 3-chloro-1,2-dithiolane (focused on potential energy terms and symmetry considerations) can be found in ref (33) (with regard to 1,2-dioxolane, see also the earlier contributions of Cremer).^14,15^ Unless otherwise specified, the mass of the most abundant isotope of the element is assumed for each atom. The computational data provided in this section allow a direct comparison with the experimental observables, especially with regard to experimental transition frequencies measured with far-infrared spectroscopies. An indirect comparison with experimental transition energies measured in the microwave region (i.e., rotational transition energies) is possible as well: in this case, *differences* of ν-specific rotational constants should be compared with differences of experimental rotational constants associated with different pseudorotational eigenstates.

### 4.1 1D-PESs, Eigenvalues, and Eigenvectors of *Ĥ*_vib_^0^

**Figure 4:**
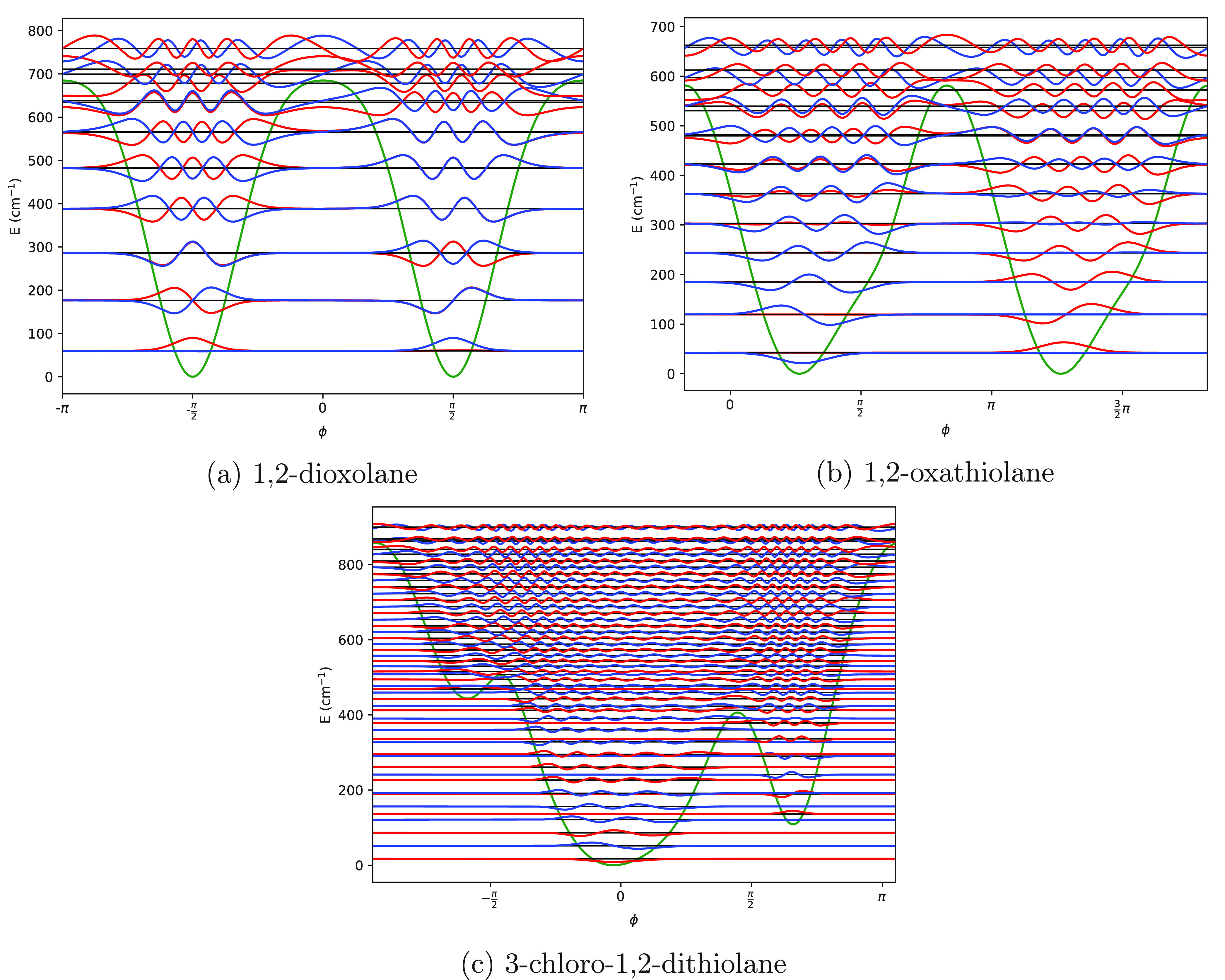
Solutions of the 1D-NSE for the case *J* = 0. The 1D-PES is depicted with a continuous green line. Eigenvalues are labeled with horizontal black lines, while the corresponding eigenvectors are represented with red and blue lines (the red color is employed for eigenstates with pair indices, i.e. ν = 0, 2, 4, ..., while the blue color is adopted for the other ones, i.e. ν = 1, 3, ...).

Eigenvalues and eigenvectors of *Ĥ*_vib_^0^ are shown in Figure 4 for each of the molecular systems investigated in this work. Moreover, 1D-PESs are shown: in this manner, the effects of *V*(ϕ) (i.e., magnitude and shapes of the potential energy barriers) on eigenvalues and eigenfunctions are highlighted.

The 1D-PESs associated with the pseudorotation angle ϕ exhibit a symmetry which is correlated to the symmetry of the planar configuration of a 5-term ring system (this fact was explicitly pointed out by Cremer and co-workers^14,36^ and further explored in ref (33)). More specifically, in the case of 1,2-dioxolane, whose planar configuration pertains to the *C*_2*v*_ symmetry point group, the potential energy term satisfies both *V*(ϕ) = *V*(ϕ + π) and *V*(ϕ) = *V*(−ϕ); therefore, the 1D-PES can be fitted with a Fourier series containing only the cosine terms of the even harmonics. With regard to 1,2-oxathiolane (*C*_*s*_), only the relation *V*(ϕ) = *V*(ϕ + π) is satisfied and the 1D-PES can be fitted with a Fourier series containing both the sine and cosine terms of the even harmonics.^33^ Finally, the planar configuration of 3-chloro-1,2-dithiolane pertains to the *C*_1_ symmetry point group; therefore, symmetry cannot be employed to simplify the picture. However, the physical nature of the pseudorotational motion (and the mathematical definition of ϕ) ensures the periodicity of the 1D-PES for each of the molecular systems investigated in this work, i.e., *V*(ϕ) = *V*(ϕ + 2π) in all the cases (also in the case of 3-chloro-1,2-dithiolane).

**Figure 5:**
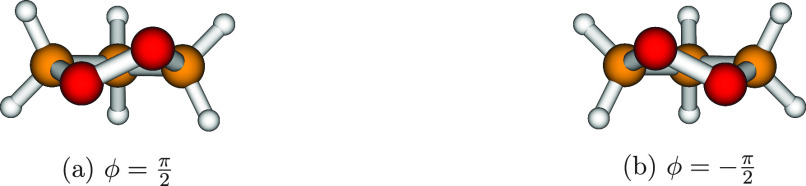
Optimized structures of 1,2-dioxolane. The two geometries are referred to the minima of the 1D-PES depicted in Figure 4a.

**Figure 6:**
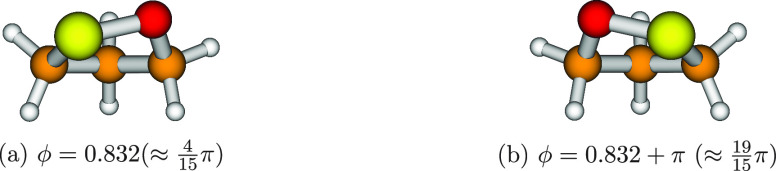
Optimized structures of 1,2-oxathiolane. The two geometries are referred to the minima of the 1D-PES depicted in Figure 4b.

**Figure 7:**
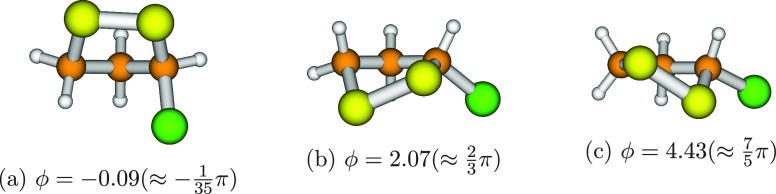
Optimized structures of 3-chloro-1,2-dithiolane. The three geometries are referred to the minima of the 1D-PES depicted in Figure 4c.

The two minima of the 1D-PES shown in Figure 4a correspond to the same value of the potential energy and to two different structures; these structures form an enantiomeric pair, shown in Figure 5. A similar picture holds in the case of 1,2-oxathiolane: the optimized structures (which correspond to the two minima of the 1D-PES displayed in Figure 4b) are enantiomers and are depicted in Figure 6. The less symmetric 1D-PES of 3-chloro-1,2-dithiolane (see Figure 4c) is characterized by three minima with different energies and structures (reported in Figure 7).

Turning to the solutions of the 1D-NSE, Figure 4 deserves some comments. First, the eigenfunctions plotted in Figure 4 are not normalized, and the zeroes of their amplitudes correspond to the numerical value of the specific eigenvalue associated with the eigenfunction of interest. Second, the sign of an eigenfunction is not relevant, i.e. ψ_ν_ and – ψ_ν_ (where ν labels the eigenstate) denotes exactly the same eigenfunction (this statement is rather obvious when the eigenfunctions are not normalized, i.e., defined up to a numerical constant; however, the statement is true also for normalized eigenfunctions). Finally, it must be pointed out that when two eigenvalues are almost degenerate, their corresponding eigenfunctions can be partially superimposed in Figure 4, and therefore, one of the two can be partially hidden from the other one (e.g., this is the case of the eigenfunctions ψ_4_ and ψ_5_ of 1,2-dioxolane).

Eigenvalues of *Ĥ*_vib_^0^ (labeled with *E*_ν_) are reported in Tables 1, 2, and 3 in order to allow quantitative comparisons. The patterns of *E*_ν_ reported in Tables 1 (1,2-dioxolane) and 2 (1,2-oxathiolane) are quite similar: these results can be explained as a consequence of similarities concerning structural features and potential energy profiles. With regard to the structures, the only difference between the two molecules concerns the substitution of one of the two oxygen atoms of 1,2-dioxolane with the sulfur atom found in 1,2-oxathiolane. This substitution increases the mass of one of the atoms directly involved in the central 5-term ring, leading to an increase of the density of states: in the energy interval 0–500 cm^–1^, there are 10 eigenstates in the case of 1,2-dioxolane (see Figure 4a and Table 1) and 16 eigenstates in the case of 1,2-oxathiolane (see Figure 4b and Table 2). Moreover, the substitution removes one of the symmetry elements of 1,2-dioxolane, with the consequences already mentioned on the symmetry of the 1D-PESs (symmetric and asymmetric shapes of the potential energy barriers in the cases of 1,2-dioxolane and 1,2-oxathiolane, respectively; see Figure 4a,b).

**Table 1:** Eigenvalues of *Ĥ*_vib_^0^ (*E*_ν_), Lowest Rotational Transition Energies (*J* = 0 → *J* = 1), and Rotational Constants (*A*_ν_, *B*_ν_, and *C*_ν_), for Each Eigenstate of the 1D-NSE Associated to the Pseudorotational Motion of 1,2-Dioxolane

ν	*E*_ν_ (cm^–1^)	Δ*E*_ν_[1_01_] (MHz)	Δ*E*_ν_[1_11_] (MHz)	Δ*E*_ν_[1_10_] (MHz)	*A*_ν_ (MHz)	*B*_ν_ (MHz)	*C*_ν_ (MHz)
0	59.84	11529.3	11709.5	14855.5	7517.9	7337.6	4191.6
1	59.84	11532.4	11712.7	14855.5	7517.9	7337.6	4194.8
2	176.42	11521.7	11711.3	14852.8	7521.2	7331.6	4190.1
3	176.42	11517.1	11706.7	14852.8	7521.2	7331.6	4185.5
4	286.15	11506.3	11704.7	14850.1	7524.3	7325.9	4180.4
5	286.15	11512.0	11710.5	14850.1	7524.3	7325.8	4186.2
6	388.36	11497.5	11704.4	14847.4	7527.2	7320.3	4177.3
7	388.36	11497.7	11704.6	14847.4	7527.2	7320.2	4177.4
8	482.24	11488.4	11704.0	14844.8	7530.2	7314.6	4173.8
9	482.26	11487.9	11704.0	14844.3	7530.2	7314.1	4173.8
10	565.74	11475.2	11700.8	14838.9	7532.2	7306.7	4168.6
11	566.07	11476.2	11700.0	14840.2	7532.0	7308.2	4168.0
12	634.07	11462.2	11704.9	14835.5	7539.1	7296.4	4165.8
13	638.02	11458.2	11694.9	14836.8	7536.7	7300.1	4158.2
14	678.42	11453.6	11719.1	14831.3	7548.4	7282.9	4170.7
15	699.47	11376.8	11623.8	14834.7	7540.8	7293.9	4082.9
16	710.91	11524.6	11793.2	14831.5	7550.1	7281.4	4243.2

**Table 2:** Eigenstate Indices of the 1D-NSE Associated to the Pseudorotational Motion of 1,2-Oxathiolane (ν), Eigenvalues of *Ĥ*_vib_^0^ (*E*_ν_), Lowest Rotational Transition Energies (*J* = 0 → *J* = 1), and Rotational Constants (*A*_ν_, *B*_ν_, and *C*_ν_)

ν	*E*_ν_ (cm^–1^)	Δ*E*_ν_[1_01_] (MHz)	Δ*E*_ν_[1_11_] (MHz)	Δ*E*_ν_[1_10_] (MHz)	*A*_ν_ (MHz)	*B*_ν_ (MHz)	*C*_ν_ (MHz)
0	42.23	7695.1	9364.7	11123.0	6396.3	4726.7	2968.4
1	42.23	7695.1	9364.7	11123.0	6396.3	4726.7	2968.4
2	119.53	7697.6	9366.2	11117.9	6393.3	4724.7	2972.9
3	119.53	7697.6	9366.2	11117.9	6393.3	4724.7	2972.9
4	184.78	7703.1	9370.1	11113.7	6390.4	4723.3	2979.7
5	184.78	7703.0	9370.1	11113.7	6390.4	4723.3	2979.7
6	243.78	7706.9	9374.0	11112.4	6389.8	4722.6	2984.2
7	243.79	7706.8	9374.0	11112.4	6389.8	4722.6	2984.2
8	303.01	7707.0	9376.4	11112.9	6391.2	4721.7	2985.2
9	303.03	7707.0	9376.4	11112.9	6391.2	4721.7	2985.2
10	363.08	7705.6	9378.4	11113.5	6393.2	4720.4	2985.3
11	363.17	7705.0	9379.0	11113.4	6393.7	4719.7	2985.3
12	422.68	7703.2	9380.4	11113.6	6395.3	4718.2	2985.0
13	423.10	7703.0	9379.7	11113.8	6395.3	4718.6	2984.4
14	479.74	7701.3	9384.1	11114.9	6398.8	4716.1	2985.3
15	481.89	7698.6	9379.6	11114.6	6397.8	4716.8	2981.8
16	530.46	7700.9	9391.7	11116.7	6403.7	4713.0	2988.0
17	539.47	7687.4	9372.1	11115.5	6400.1	4715.4	2972.0
18	572.10	7706.8	9404.4	11118.1	6407.8	4710.3	2996.6
19	597.24	7641.0	9327.6	11116.1	6401.3	4714.7	2926.3
20	612.23	7750.5	9446.8	11118.0	6407.1	4710.9	3039.7

**Table 3:** 3-Chloro-1,2-dithiolane: Eigenvalues of *Ĥ*_vib_^0^ (*E*_ν_), Lowest Rotational Transition Energies (*J* = 0 → *J* = 1), and ν-Specific Rotational Constants (*A*_ν_, *B*_ν_, and *C*_ν_)

ν	*E*_ν_ (cm^–1^)	Δ*E*_ν_[1_01_] (MHz)	Δ*E*_ν_[1_11_] (MHz)	Δ*E*_ν_[1_10_] (MHz)	*A*_ν_ (MHz)	*B*_ν_ (MHz)	*C*_ν_ (MHz)
0	17.15	2670.8	4290.7	4543.5	3081.7	1461.8	1209.0
1	51.65	2675.5	4299.4	4550.1	3087.0	1463.1	1212.4
2	86.35	2674.9	4313.2	4562.4	3100.4	1462.0	1212.8
3	121.26	2671.2	4329.9	4577.9	3118.3	1459.6	1211.6
4	136.08	2486.5	4447.5	4672.5	3316.7	1355.7	1130.8
5	156.38	2665.9	4346.7	4593.8	3137.3	1456.5	1209.4
6	189.79	2498.9	4439.2	4663.0	3301.6	1361.4	1137.5
7	191.53	2660.4	4363.3	4609.5	3156.2	1453.3	1207.1
8	226.51	2655.2	4378.6	4623.9	3173.6	1450.3	1205.0
9	241.20	2512.8	4430.4	4653.0	3285.3	1367.7	1145.1
10	261.14	2650.3	4392.8	4637.2	3189.8	1447.4	1203.0
11	290.13	2529.1	4419.3	4640.4	3265.3	1375.1	1154.0
12	295.19	2646.8	4404.7	4648.1	3203.0	1445.1	1201.7
13	328.35	2644.7	4414.0	4655.9	3212.6	1443.3	1201.4
14	336.25	2549.9	4404.2	4623.3	3238.8	1384.5	1165.4
15	360.23	2646.0	4417.8	4657.5	3214.6	1442.8	1203.2
16	378.50	2586.8	4377.0	4593.1	3191.6	1401.5	1185.4
17	390.32	2645.3	4417.7	4653.9	3213.1	1440.8	1204.6
18	412.21	2648.8	4364.5	4584.7	3150.2	1434.5	1214.3
19	423.11	2597.8	4437.8	4669.0	3254.5	1414.5	1183.3
20	442.87	2611.6	4433.1	4663.3	3242.4	1420.9	1190.7
21	459.34	2581.2	4463.2	4695.2	3288.6	1406.6	1174.6
22	468.93	2306.6	4938.2	5199.9	3915.7	1284.2	1022.4
23	477.10	2567.8	4494.7	4730.1	3328.5	1401.6	1166.2
24	494.42	2543.0	4535.6	4773.1	3382.8	1390.3	1152.7
25	507.71	2422.6	4749.5	4999.9	3663.4	1336.6	1086.1
26	515.41	2461.4	4673.8	4920.2	3566.4	1353.9	1107.5
27	529.46	2497.3	4611.0	4853.8	3483.8	1370.0	1127.2
28	543.61	2481.9	4636.1	4880.5	3517.3	1363.2	1118.8
29	557.66	2483.9	4629.6	4873.8	3509.8	1364.1	1119.8
30	572.56	2488.0	4620.6	4864.5	3498.6	1365.9	1122.0
31	588.04	2487.8	4619.3	4863.3	3497.4	1365.9	1121.9
32	603.87	2486.9	4619.1	4863.3	3497.8	1365.5	1121.4
33	620.08	2485.9	4619.2	4863.5	3498.4	1365.1	1120.8
34	636.59	2484.7	4619.8	4864.4	3499.8	1364.6	1120.1
35	653.40	2482.8	4621.5	4866.3	3502.5	1363.8	1119.0
36	670.43	2480.9	4623.5	4868.6	3505.6	1363.0	1117.9
37	687.67	2478.4	4626.2	4871.6	3509.7	1362.0	1116.5
38	705.05	2475.8	4629.5	4875.3	3514.5	1360.8	1115.0
39	722.56	2472.7	4632.8	4879.0	3519.5	1359.5	1113.2
40	740.11	2469.3	4639.0	4885.7	3527.7	1358.0	1111.3
41	757.73	2465.6	4639.5	4886.7	3530.3	1356.4	1109.2
42	775.14	2460.4	4657.6	4905.5	3551.4	1354.2	1106.2
43	792.82	2457.0	4635.4	4883.7	3531.1	1352.6	1104.4
44	809.27	2445.9	4712.6	4962.6	3614.7	1348.0	1097.9
45	827.60	2446.8	4556.9	4806.0	3458.1	1347.9	1098.8
46	840.42	2421.0	4884.7	5138.4	3801.0	1337.4	1083.6

Despite these differences, the undeniable similarities concerning the height of the barrier to pseudorotation (slightly less than 600 and slightly less than 700 cm^–1^ for 1,2-oxathiolane and 1,2-dioxolane, respectively; see Figure 4a,b) and the two minima (which are enantiomers corresponding to the same energy values; see Figures 5 and 6) leads to a pattern which was already observed by other authors in the results obtained for other 5-term ring systems.^60,61^ Three regions can be recognized. In the first one (*E*_ν_ ≲ max[*V*(ϕ)]), pairs of almost degenerate eigenvalues are obtained (from ν = 0 to ν = 13 in the case of 1,2-dioxolane and from ν = 0 to ν = 17 in the case of 1,2-oxathiolane; see Tables 1 and 2). In the second one (*E*_ν_ ≈ max[*V*(ϕ)]), a triplet of eigenvalues is found (ν = 14, 15, 16 in Table 1; ν = 18, 19, 20 in Table 2). Finally, in the third region (*E*_ν_ > max[*V*(ϕ)]), the solutions are distributed as pairs of almost degenerate eigenvalues (these solutions are not reported in Tables 1 and 2, but the pairs ν = 17, 18 for 1,2-dioxolane and ν = 21, 22 for 1,2-oxathiolane are depicted in panels a and b of Figure 4, respectively). Moreover, in the first region (*E*_ν_ ≲ max[*V*(ϕ)]), the energy difference between two almost degenerate eigenvalues increases with the eigenvalue index (e.g., in the case of 1,2-dioxolane the energy difference between *E*_8_ and *E*_9_ is equal to 0.02 cm^–1^, while the energy difference between *E*_10_ and *E*_11_ is 0.33 cm^–1^; with regard to the pairs ν = 0, 1, ν = 2, 3, ν = 4, 5, and ν = 6, 7, the values of the energies are equal as a consequence of the limited number of digits reported in the second column of Table 1; that is, the energy differences are smaller than 0.01 cm^–1^).

The eigenvalues of *Ĥ*_vib_^0^ in the case of 3-chloro-1,2-dithiolane (second column of Table 3) exhibit a pattern which is quite different with respect to the cases discussed above (1,2-dioxolane and 1,2-oxathiolane). First, the density of states in the case of 3-chloro-1,2-dithiolane is clearly the highest one among the molecular systems investigated in this work (compare the results shown in Figure 4c with the results depicted in Figure 4a,b); this observation can be explained by the higher molecular mass of 3-chloro-1,2-dithiolane (with respect to 1,2-dioxolane and 1,2-oxathiolane), which is partly due to the presence of a chlorine atom. Second, in the region *E*_ν_ ≲ max[*V*(ϕ)] there is not a pattern of almost degenerate eigenvalues discussed above for the cases of 1,2-dioxolane and 1,2-oxathiolane (compare the values provided in the second column of Table 3 with the values reported in Tables 1 and 2). This is not surprising, because in this case there are three minima with different energies (corresponding to three asymmetric structures; see Figures 4c and 7) and a more complex potential energy profile (see Figure 4). On the other hand, when *E*_ν_ > max[*V*(ϕ)] the patterns found for the three molecular systems investigated in this work are similar, with pairs of almost degenerate eigenvalues (only the first pair of eigenstates of this region is depicted in Figure 4; the eigenstates with higher eigenvalues are not reported).

From the mathematical point of view, the 1D-NSE associated with the pseudorotation in a 5-term ring system in the *J* = 0 case is a Sturm–Liouville problem. For completeness, a concise treatment based on this alternative point of view is provided in section 3 of the Supporting Information; more specifically, the existence of exact and approximated analytical solutions to the problem investigated in this subsection is discussed, particularly with regard to the dependence of *g*^*ϕϕ*^, *V*′, and *V* (see eq 10) on the pseudorotation angle ϕ. An assessment of the importance of the structural relaxation effect (i.e., the importance of an explicit account of the dependence of *g*^*ϕϕ*^ and *V*′ on ϕ) for the solution of the 1D-NSE in the *J* = 0 case is also proposed.

### 4.2 Rotational Constants and Rotational Transition Energies

A preliminary step which is needed to construct Tables 1–3 is the assignment of a triplet of eigenstates of *Ĥ*_vibrot_^1^ to a specific eigenstate of *Ĥ*_vib_^0^. The assignment is not always possible, because its meaningfulness depends on the separability of pseudorotational and rotational motions (i.e., the approximation ψ_vibrot_^1(*x*)^ ≈ ψ_vib_^0(ν)^ψ_rot_^1(*νl*)^ must be a suitable one).

To automate the assignment, a suitable computational criterion must be selected. A simple solution can be based on the numerical values of the eigenvalues of *Ĥ*_vib_^0^ and *Ĥ*_vibrot_^1^: eigenvalues of *Ĥ*_vib_^0^ and *Ĥ*_vibrot_^1^ can be listed in ascending order (on the basis of their numerical value), and the assignment could be carried out automatically. That is, the first element of the list of eigenvalues of *Ĥ*_vib_^0^ (*E*_0_) is associated with the first, the second, and the third elements of the list of eigenvalues of *Ĥ*_vibrot_^1^ (i.e., these three elements are labeled *E*_0_[1_01_], *E*_0_[1_11_], and *E*_0_[1_10_]); the second element of the list of eigenvalues of *Ĥ*_vib_^0^ (*E*_1_) is associated with the fourth, the fifth, and the sixth elements of the other list, and so on. In practice, this solution works only if the energy differences among the eigenvalues of *Ĥ*_vib_^0^ are sufficiently large. In this work, this solution works in the case of 3-chloro-1,2-dithiolane but not in the cases of 1,2-dioxolane and 1,2-oxathiolane. Therefore, in this work another scheme was employed, which is based on the superposition of the eigenstates of *Ĥ*_vibrot_^1^ and *Ĥ*_vib_^0^ (a more detailed explanation can be found in section 2 of the Supporting Information, see eqs SI-70–SI-73): because ⟨ψ_vib_^0(ν)^|ψ_vib_^0(ν^′^)^⟩ = 0 when ν ≠ ν′, if the approximation ψ_vibrot_^1(*x*)^ ≈ ψ_vib_^0(ν)^ψ_rot_^1(*νl*)^ holds (i.e., ψ_vibrot_^1(*x*)^ ≡ ψ_vibrot_^1(*νl*)^), the superposition between ψ_vibrot_^1(*x*)^ and ψ_vib,ext._^0(ν)^ (ψ_vib,ext._^0(ν)^ is the extension of ψ_vib_^0(ν)^ to the rotational basis; for a rigorous definition, see eq SI-71 of the Supporting Information) is zero for all the values of the index ν except the one to which ψ_vibrot_^1(*x*)^ must be assigned (⟨ψ_vib,ext._^0(ν)^|ψ_vibrot_^1(*x*)^⟩ ≈ ⟨ψ_vib,ext._^0(ν)^|ψ_vib_^0(ν′)^ψ_rot_^1(ν^′^*l*)^⟩ = 0 if ν ≠ ν′). This solution is expected to work if the approximation ψ_vibrot_^1(*x*)^ ≈ ψ_vib_^0(ν)^ψ_rot_^1(*νl*)^ holds; however, if the approximation is not valid, the assignment itself is meaningless.

**Figure 8:**
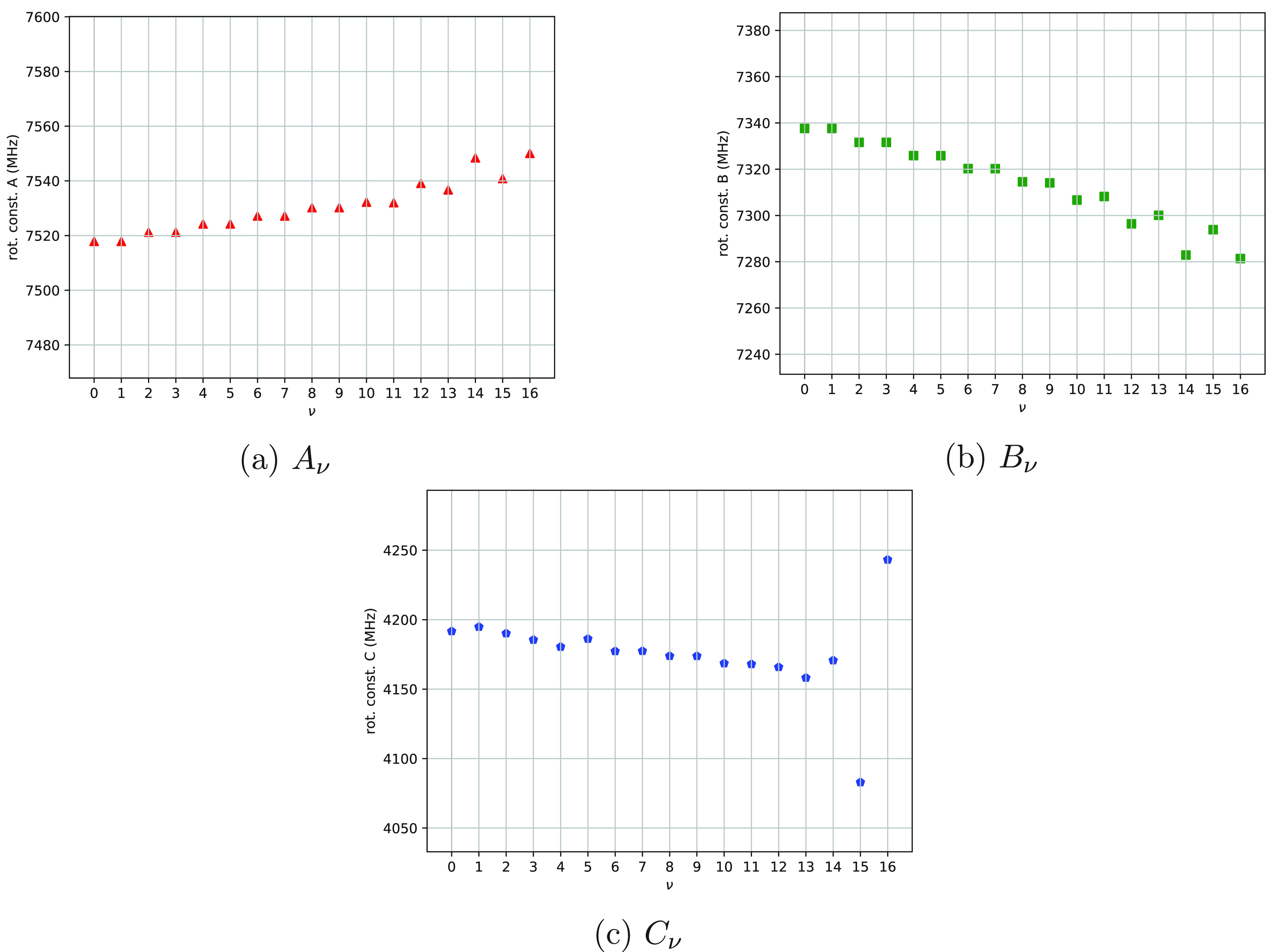
Rotational constants of 1,2-dioxolane as functions of the index ν.

**Figure 9:**
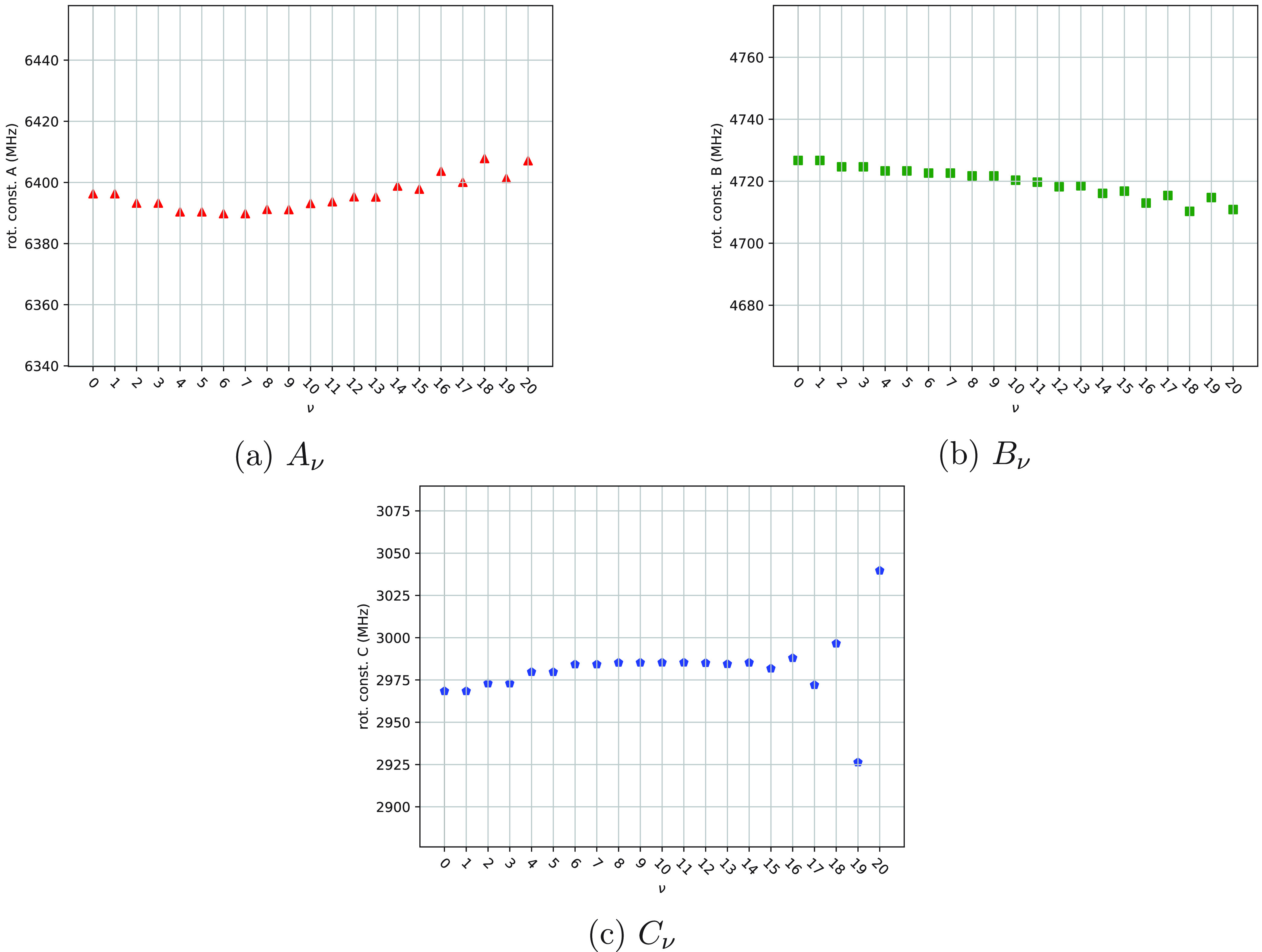
Rotational constants of 1,2-oxathiolane as functions of the index ν.

**Figure 10:**
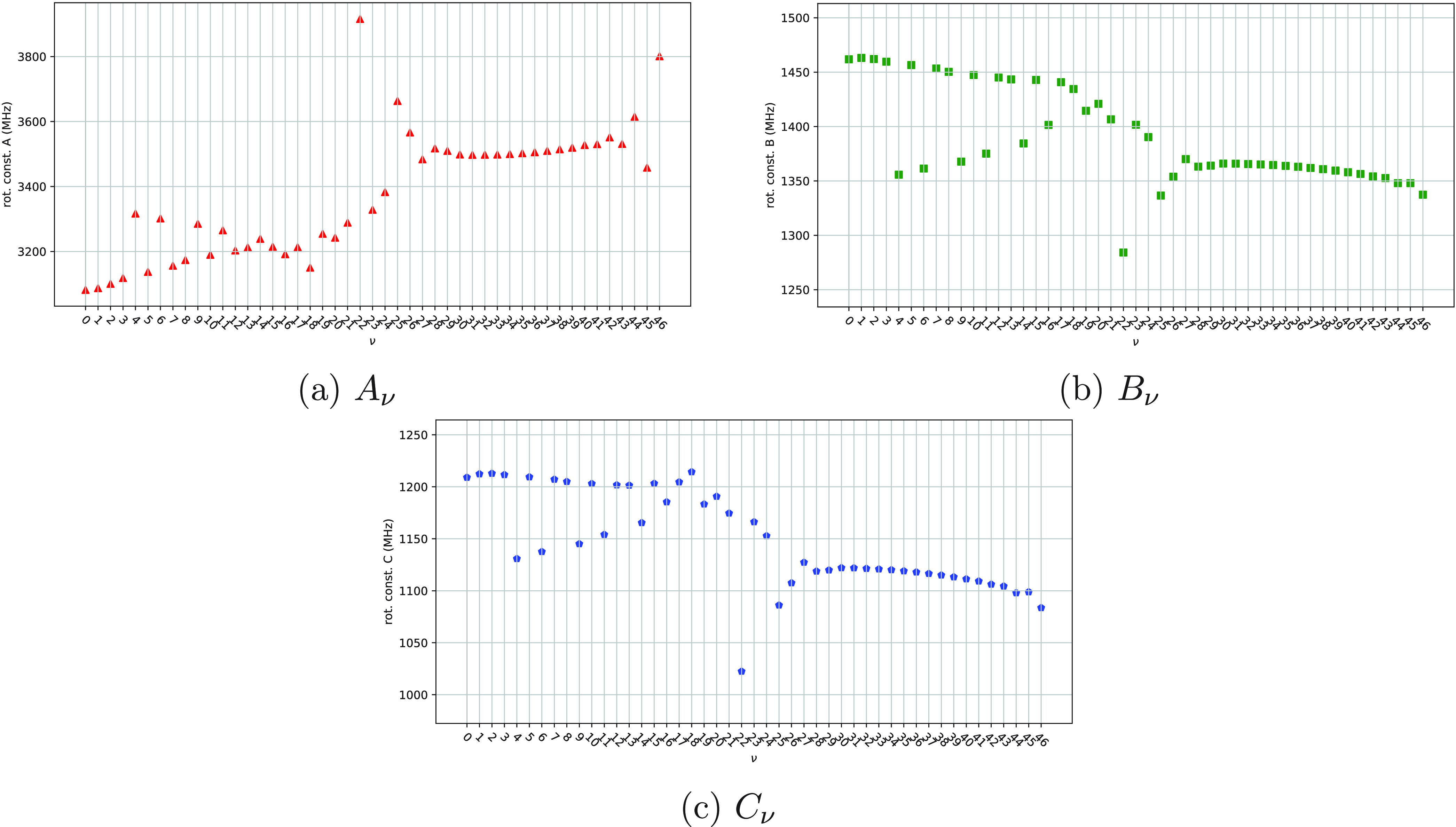
Rotational constants of 3-chloro-1,2-dithiolane as functions of the index ν.

For each eigenstate of *Ĥ*_vib_^0^ reported in Tables 1–3, the eigenvalue is subtracted from the three eigenvalues of *Ĥ*_vibrot_^1^ assigned to the eigenstate: in this manner, the three lowest rotational transition energies (Δ*E*_ν_[1_01_] = *E*_ν_[1_01_] – *E*_ν_, Δ*E*_ν_[1_11_] = *E*_ν_[1_11_] – *E*_ν_, and Δ*E*_ν_[1_10_] = *E*_ν_[1_10_] – *E*_ν_) are calculated. From these values the rotational constants *A*_ν_, *B*_ν_, and *C*_ν_ can be obtained because
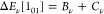
12
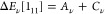
13
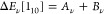
14The rotational constants *A*_ν_, *B*_ν_, and *C*_ν_ are plotted as functions of the index ν in Figures 8–10. Values of the equilibrium rotational constants *A*_e_, *B*_e_ and *C*_e_ for the optimized geometries (see Figures 5–7) of the three molecular systems investigated in this work are provided in Table 4.

**Table 4:** Equilibrium Rotational Constants of the Optimized Geometries of the Molecular Systems Investigated in This Work

structure	*A*_e_	*B*_e_	*C*_e_	figure
1,2-dioxolane^a^	7516.4	7340.7	4195.4	5a,b
1,2-oxathiolane^a^	6397.6	4728.0	2967.2	6a,b
3-chloro-1,2-dithiolane I^b^	3081.4	1460.6	1206.6	7a
3-chloro-1,2-dithiolane II^b^	3324.5	1352.9	1127.5	7b
3-chloro-1,2-dithiolane III^b^	3914.4	1283.4	1021.6	7c

a Only one set of values is reported because two enantiomers have the same equilibrium rotational constants.

b The roman numerals refer to the energies (labeled in ascending order).

As mentioned in the previous subsection, in the cases of 1,2-dioxolane and 1,2-oxathiolane the eigenvalues of the first eigenstates of *Ĥ*_vib_^0^ are distributed in pairs of almost equal numerical values. Therefore, eigenvalues of the eigenstates of *Ĥ*_vibrot_^1^ which are assigned to two almost degenerate eigenstates of *Ĥ*_vib_^0^ are expected to be very similar. This is the case of 1,2-oxathiolane: the numerical values of Δ*E*_ν_[1_01_], Δ*E*_ν_[1_11_], and Δ*E*_ν_[1_10_] reported in Table 2 are equal (or almost equal) for each of the following pairs of indices: ν = 0, 1; ν = 2, 3; ν = 4, 5; ν = 6, 7; and ν = 8, 9. The increment of the energy difference between two almost degenerate eigenvalues of *Ĥ*_vib_^0^ is parallel to the increase of the numerical differences of the values of Δ*E*_ν_[1_01_], Δ*E*_ν_[1_11_], and Δ*E*_ν_[1_10_] assigned to the two eigenvalues of *Ĥ*_vib_^0^; this is the case of the pairs ν = 14, 15 and ν = 16, 17 (see the values reported in Table 2). Numerical values of *A*_ν_, *B*_ν_, and *C*_ν_ exhibit the same regularities (see Figure 9) mentioned for the values of Δ*E*_ν_[1_01_], Δ*E*_ν_[1_11_], and Δ*E*_ν_[1_10_] (this is obvious, taking into account eqs 12–14). However, these regularities do not involve the eigenvalues of *Ĥ*_vibrot_^1^ assigned to the triplet of eigenstates of *Ĥ*_vib_^0^ labeled with ν = 18, 19, 20; the relevant variations are particularly evident in the plot of *C*_ν_ values (Figure 9c) and are due to the numerical values of Δ*E*_ν_[1_01_] and Δ*E*_ν_[1_11_] (see Table 2) . Despite the undeniable dependence of *A*_ν_, *B*_ν_, and *C*_ν_ on the value of the index ν, in the case of 1,2-oxathiolane limited changes from the original equilibrium values (*A*_e_, *B*_e_, and *C*_e_) are observed between ν = 0 and ν = 17 (see Tables 2 and 4): values of *A*_ν_ are in the interval 6389–6404 MHz (with a value of *A*_e_ of 6397.6 MHz), values of *B*_ν_ in the interval 4713–4727 MHz (with *B*_e_ equals to 4728.0 MHz), and values of *C*_ν_ in the interval 2968–2988 MHz (with *C*_e_ = 2967.2 MHz).

With regard to 1,2-dioxolane, rotational constants are plotted in Figure 8. The analysis proposed above for the 1,2-oxathiolane molecule is partially valid also for 1,2-dioxolane. However, some differences must be pointed out. First, the values of Δ*E*_ν_[1_10_], *A*_ν_ and *B*_ν_ assigned to a pair of almost degenerate eigenstates of *Ĥ*_vib_^0^ are equal (or almost equal; see Table 1 and Figure 8a,b) for the lowest ν values (analogous to the case of 1,2-oxathiolane), while the values of Δ*E*_ν_[1_01_], Δ*E*_ν_[1_11_], and *C*_ν_ assigned to the same pairs of almost degenerate eigenstates of *Ĥ*_vib_^0^ are different (see Table 1 and Figure 8c) even in the case of the pair ν = 0, 1 (in contrast with the case of 1,2-oxathiolane). Second, the values of *A*_ν_, *B*_ν_, and *C*_ν_ range in the intervals 7517–7539, 7296–7338, and 4158–4194, respectively (with *A*_e_ = 7516.4, *B*_e_ = 7340.7, and *C*_e_ = 4195.4; see Tables 1 and 4); these intervals are wider than the ones calculated for 1,2-oxathiolane.

The pattern shown in Figure 8c is not the expected one (at least for ν values in the range between 0 and 5) and deserves further comments. In the case of 1,2-dioxolane, the reference system adopted for the Cartesian framework of each optimized molecular structure might be inadequate: as a consequence, the pattern calculated for Δ*E*_ν_[1_01_], Δ*E*_ν_[1_11_], and *C*_ν_ might be incorrect. As mentioned in Computational Protocol, all the results proposed in this section were calculated employing the PAS reference system. To verify the reliability of the results proposed in Table 1 (and shown in Figure 8) the Hamiltonians *Ĥ*_vib_^0^ and *Ĥ*_vibrot_^1^ were computed employing another reference system, named the zeta axis system (ZAS). ZAS is described in section 4 of the Supporting Information, where a comparison of the results obtained with the two reference systems (PAS and ZAS) is proposed. When the ZAS reference system is employed, the expected patterns (first eigenvalues distributed in pairs of equal numerical values) of Δ*E*_ν_[1_01_], Δ*E*_ν_[1_11_], and *C*_ν_ are obtained (see Table SI 4 and Figure SI 6). Therefore, a possible explanation for the discrepancies between the values of Δ*E*_ν_[1_01_], Δ*E*_ν_[1_11_], and *C*_ν_ reported in Table 1 and the expected ones is related to the choice of the reference axis system.

**Figure 11:**
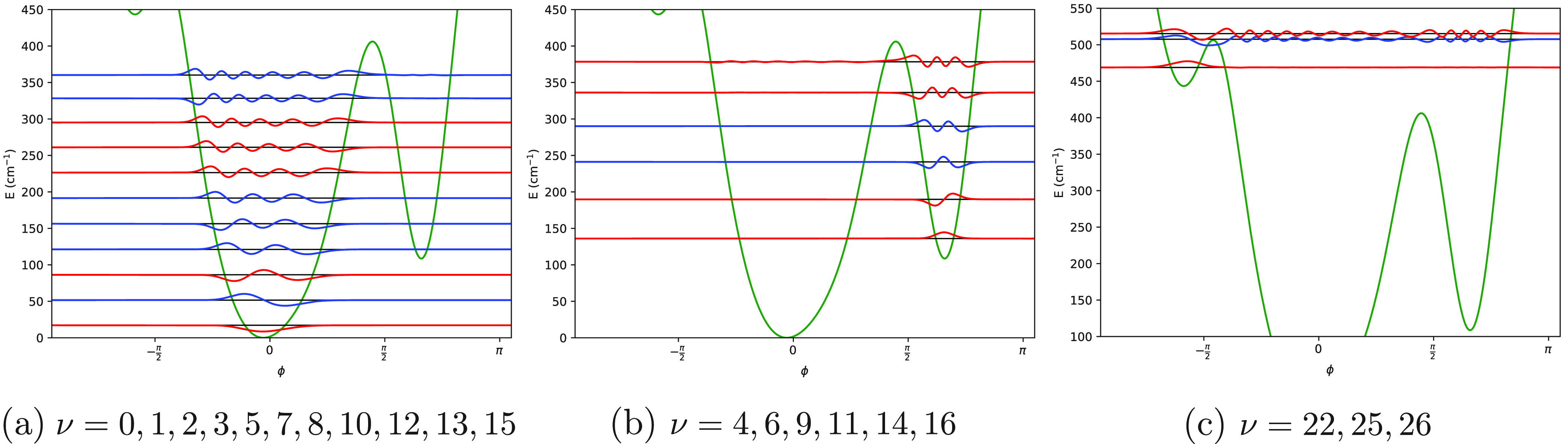
1D-PES (continuous green line) and a selection of eigenvalues (horizontal black line) and eigenvectors (red for the eigenstates with pair indices, blue for eigenstates with odd indices) of *Ĥ*_vib_^0^ reported for 3-chloro-1,2-dithiolane molecular system. Each subfigure is dedicated to one of the three convergent branches depicted in panels a, b, and c of Figure 10 (for more details, see the text). More specifically, panels a, b, and c are referred to the first, second, and third branch, respectively. The eigenvectors depicted in this figure are not normalized.

The results obtained for 3-chloro-1,2-dithiolane are reported in Table 3 and shown in Figure 10; these results are qualitatively different from the ones depicted in Figures 8 and 9. From lower to higher values of the index ν, three distinct and convergent branches can be identified in each plot (see Figure 10a–c): The first branch consists of the rotational constants corresponding to the following values of the index ν: 0, 1, 2, 3, 5, 7, 8, 10, 12, 13, and 15. The second one comprises the rotational constants corresponding to the values 4, 6, 9, 11, 14, and 16 of the index ν. The third (and the shortest) one involves the rotational constants corresponding to the values 22, 25, and 26 of the index ν. The three branches can be associated with the three minima of the 1D-PES shown in Figure 4c because the values of the ν index, which identify one of the three branches, correspond to eigenstates of *Ĥ*_vib_^0^ which are prevalently located in the potential well of one of the three minima. This is particularly evident in the cases of the first and the second branches; for the sake of clarity, the relevant portion of the 1D-PES and the eigenstates of *Ĥ*_vib_^0^ corresponding to a specific branch of Figure 10a–c are shown in Figure 11. With regard to the third branch, it must be underlined that only one eigenstate is prevalently located inside the potential well: this is not surprising and is due to the shallowness of the potential well associated with this branch (see Figure 11c). However, the amplitude of the other two eigenstates corresponding to the third branch is clearly affected by the highest potential well of Figure 11c, and the results presented so far suggest an influence of the highest potential well of Figure 11c also for the values of the rotational constants assigned to the eigenstates 25 and 26. The rotational constants assigned to the first eigenstate of each of the three branches (see the rotational constants associated to the eigenstates 0, 4, and 22 in Table 3) are close to the equilibrium rotational constants reported in Table 4 (associated to the optimized geometries of Figure 7).

## 5 Accuracy, Limitations, and Possible Improvements of the Model

Some limitations of the model employed in this work can be readily identified taking into account the alternative approaches mentioned in the Introduction. When the computed potential energy barriers are extremely small (which is not the case of the molecules investigated in this work), SAMs can affect the final results of the 1D problem even qualitatively (in the case of 1,3-dioxolane molecule this aspect was taken into account in ref (62); see refs (63 and 64) for a comparison with experimental results). In this case, the effects of SAMs on the calculated solutions of the nuclear problem cannot be safely neglected. Moreover, to reproduce the numerical values of experimental rotational constants the interactions of the molecular rotations with *all* the internal degrees of freedom (and not only with the pseudorotational motion) must be taken into account.

To carefully evaluate the usefulness of the computational results proposed in the previous section, an estimation of their accuracy should be provided. To benchmark the computational results reported in the previous section a comparison with the results of experimental measurements performed on 1,2-dioxolane, 1,2-oxathiolane, and 3-chloro-1,2-dithiolane is needed. To the best of the authors’ knowledge, suitable experimental data are not available in the literature for 1,2-oxathiolane and 3-chloro-1,2-dithiolane. In the case of 1,2-dioxolane, ground-state rotational constants are reported in ref (65). With the available data, only a rough estimation of the accuracy is possible.

The accuracy of the energy values employed to construct the 1D-PESs (see Figure 4) depends on the level of theory chosen for the electronic structure calculations (in this work, B3LYP/maug-cc-pVTZ). In ref (33), the same basis set was employed in conjunction with a double hybrid exchange–correlation functional^66^ and empirical dispersions^67,68^ (B2PLYP+D3BJ/maug-cc-pVTZ); suitable experimental data were available for some of the 5-term ring systems examined in ref (33), allowing an estimation of the accuracy associated with the level of theory employed in that work, which turned out to be satisfactory (particularly for molecules with relevant pseudorotational barriers). The 1D-PESs proposed in this work are not equal, but they are similar to the results reported in ref (33) (see Table 5). Therefore, the 1D-PESs employed in this work should be at least qualitatively correct.

**Table 5:** Critical Points of the 1D-PESs: Relative Energies (with Respect to the Global Minimum, in cm^–1^) and Cremer–Pople Parameters (*q* is Provided in Angstrom) at B3LYP and B2PLYP(D3BJ) Levels

molecule	minima (*q*, ϕ, Δ*E*) B3LYP/maug-cc-pVTZ	minima (*q*, ϕ, Δ*E*) B2PLYP(D3BJ)/maug-cc-pVTZ^a^	transition states (*q*, ϕ, Δ*E*) B3LYP/maug-cc-pVTZ	transition states (*q*, ϕ, Δ*E*) B2PLYP(D3BJ)/maug-cc-pVTZ^a^
1,2-dioxolane	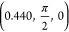	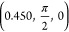	(0.389, π, 685)	(0.395, π, 721)
	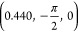	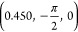	(0.389, 2π, 685)	(0.395, 2π, 721)
1,2-oxathiolane	(0.432, 0.832, 0)	(0.442, 0.817, 0)^b^	(0.432, −0.539, 581)	(0.443, – 0.528, 620)^b^
	(0.432, 0.832 + π, 0)	(0.442, 0.817 + π, 0)^b^	(0.432, – 0.539 + π, 581)	(0.443, −0.528 + π, 620)^b^
3-chloro-1,2-dithiolane	(0.456, −0.088, 0)	(0.465, 0.184, 0)	(0.587, 1.401, 407)	(0.616, 1.432, 363)
	(0.590, 2.070, 109)	(0.611, 2.027, 178)	(0.655, −1.442, 507)	(0.665, −1.404, 530)
	(0.651, −1.846, 445)	(0.668, −1.816, 448)	(0.470, −2.943, 858)	(0.479, −2.999, 973)

a Taken from ref (33).

b In this case, the pseudorotation angle ϕ reported in Table 3 of ref (33) is different: the reason is the different sequential numeration adopted for the atoms directly involved in the 1,2-oxathiolane ring (in this work, C1—C2—O3—S4—C5, while in ref (33) C1—C2—S3—O4—C5). If the same sequential numeration is employed, the same numbers are obtained (in any case, the numbers are referred to the same molecular structure, i.e., different values are due to the different convention adopted).

To estimate the accuracy of the approximated 1D flexible model employed in this work to formulate and to solve the nuclear problem, a comparison with the results of DVPT2 calculations^69^ (performed with the Gaussian 16 suite of programs) is provided in Tables 6 and 7. A perturbative treatment should be adequate for the characterization of the lowest eigenstates of the molecular systems investigated in this work; each minimum is located at the bottom of a slightly anharmonic potential energy well, and therefore, a VPT2 treatment should provide reliable results (this is no longer true when the splitting due to the tunneling between two adjacent wells is relevant).

**Table 6:** Comparison among Vibrational Transition Frequencies Calculated with Harmonic, DVPT2, and 1D Flexible Models

structure	transition^a^	harmonic model	DVPT2 model	1D flexible model
1,2-dioxolane	fundamental (0 → 2)	121.54	116.60	116.58
	overtone (0 → 4)	243.08	228.78	226.31
1,2-oxathiolane	fundamental (0 → 2)	89.17	83.17	77.30
	overtone (0 → 4)	178.34	155.41	142.55
3-chloro-1,2-dithiolane I^b^	fundamental (0 → 1)	34.78	32.68	34.50
	overtone (0 → 2)	69.56	64.83	69.20
3-chloro-1,2-dithiolane II^b^	fundamental (4 → 6)	54.04	53.33	53.71
	overtone (4 → 9)	108.09	108.43	105.12

a Numbers are referred to the eigenvalues ν calculated with the 1D flexible model (see Tables 1–3).

b The roman numerals refer to the conformational energies (labeled in ascending order).

**Table 7:** Corrections of Rotational Constants (in MHz) Due to the Interplay between Rotational and Pseudorotational Motions: Comparison between DVPT2 and 1D Flexible Models

structure	rotational constant	equilibrium value	DVPT2 model (lowest mode correction)	1D flexible model
1,2-dioxolane	*A*	7516.4	7517.5	7518.0(ν = 0)^a^
	*B*	7340.7	7337.7	7337.3(ν = 0)^a^
	*C*	4195.4	4194.6	4192.9(ν = 0)^a^
1,2-oxathiolane	A	6397.6	6396.1	6396.3(ν = 0)^b^
	B	4728.0	4726.7	4726.7(ν = 0)^b^
	C	2967.2	2968.9	2968.4(ν = 0)^b^
3-chloro-1,2-dithiolane I	A	3081.4	3083.9	3081.7(ν = 0)^b^
	B	1460.6	1461.4	1461.8(ν = 0)^b^
	C	1206.6	1208.4	1209.0(ν = 0)^b^
3-chloro-1,2-dithiolane II	A	3324.5	3324.0	3316.7(ν = 4)^b^
	B	1352.9	1353.8	1355.7(ν = 4)^b^
	C	1127.5	1128.6	1130.8(ν = 4)^b^

a Calculated employing the ZAS reference system.

b Calculated employing the PAS reference system (see Tables 1–3).

The data reported in Table 6 show a good agreement between vibrational transition energies calculated with DVPT2 and 1D flexible models and suggest the reliability of the 1D model employed in this work at least for the calculation of the differences between the lowest eigenvalues associated with the pseudorotational motion. 1D models are often employed to reproduce experimental vibrational transition energies associated with a specific LAM (see for example refs (70 and 71)): for the molecular systems characterized in this work such a comparison between computational and experimental data would be extremely useful, especially to quantitatively assess the numerical values computed for the eigenvalues of *Ĥ*_vib_^0^.

With regard to the calculation of rotational constants, it must be pointed out that a 1D model cannot be employed to calculate *all* the corrections arising from the vibration–rotation interaction, because *A*_**ν**_ ≠ *A*_ν_, *B*_**ν**_ ≠ *B*_ν_, and *C*_**ν**_ ≠ *C*_ν_ (where **ν** ≡ {ν_1_, ν_2_, *...*, ν_3*K*–6_}). In other words, the vibration–rotation interaction term due to a single mode is included in the calculated values of *A*, *B*, and *C* reported in Table 7 (namely, *A*_0_ = *A*_e_ + Δ*A*_0_), while to reproduce the ground-state experimental values *A*_**0**_, *B*_**0**_, and *C*_**0**_ the vibration–rotation interaction terms due to all the internal degrees of freedom should be included (namely, *A*_**0**_ = *A*_e_ + ∑_*i* = 1_^3*K*–6^Δ*A*_0*i*_). As a consequence, the disagreement between the calculated values of 1,2-dioxolane reported in Table 7 and the experimental values of the rotational constants taken from ref (65) is expected, because the values correspond to conceptually distinct quantities (i.e., *A*_**0**_ ≠ *A*_0_).

Corrections of the rotational constant values due to the interaction between rotational and pseudorotational motions were calculated with DVPT2 and 1D flexible models (see Table 7). The signs of the calculated corrections are the same for both the models and for each rotational constant, while some differences are observed with regard to the magnitude (especially in the case of 3-chloro-1,2-dithiolane, for which the major discrepancy between DVPT2 and 1D flexible models is found; see Table 7).

ν-specific rotational constants reported in this work could be useful to reproduce the differences between rotational constants (*A*_1_ – *A*_0_ ≈ ); this point should be verified through a comparison with experimental values of the rotational constants associated to excited pseudorotational eigenstates. Unfortunately, the experimental values of *A*_**0**_, *B*_**0**_, and *C*_**0**_ are available only for the 1,2-dioxolane molecule, and the rotational constants associated with excited pseudorotational eigenstates are not available in the literature for the three molecular systems investigated in this work.

In the case of 1,2-dioxolane, the calculated values of the equilibrium rotational constants *A*_e_, *B*_e_, and *C*_e_ are not far from the experimental values of *A*_**0**_, *B*_**0**_, and *C*_**0**_ (see Table 8). With regard to the equilibrium rotational constants, the values calculated at the B2PLYP(D3BJ)/maug-cc-pVTZ level of theory should be more accurate than the values calculated at the B3LYP/maug-cc-pVTZ level of theory (see the values reported in Table 8), as claimed by previous computational studies.^72−75^ Moreover, the calculated value of *C*_e_ obtained at the B3LYP/maug-cc-pVTZ level of theory (see Table 8) can be questioned, because calcd *C*_e_ < exptl *C*_**0**_ (generally, the inclusion of all the vibration–rotation interaction terms to perform the calculation of *A*_**0**_, *B*_**0**_, and *C*_**0**_ starting from the values of *A*_e_, *B*_e_, and *C*_e_ is expected to provide a lower value of the calculated rotational constants, i.e., *C*_**0**_ < *C*_e_). However, the computational approach suggested in this work should be employed to reproduce the *differences* between experimental rotational constants, rather than their absolute values. Accurate values of the equilibrium rotational constants are not needed if the efforts are devoted to the calculation of differences between experimental rotational constants; in this case, the accuracy of the numerical values calculated at the B3LYP/maug-cc-pVTZ level of theory should be acceptable.

**Table 8:** Equilibrium and Experimental Rotational Constants (in MHz) of 1,2-Dioxolane

molecule	rotational constant	B3LYP/maug-cc-pVTZ equilibrium value	B2PLYP(D3BJ)/maug-cc-pVTZ equilibrium value	experimental value^a^**ν** = **0**
1,2-dioxolane	*A*	7516.4	7525.1	7500.0
	*B*	7340.7	7367.9	7328.4
	*C*	4195.4	4219.2	4209.8

a Taken from ref (65).

Finally, a brief summary of the conclusions proposed in this section is provided:

*Accuracy:* For a quantitative assessment of the numerical values reported in this work, an extensive comparison with experimental data is highly desirable. However, for the *J* = 0 case, the picture provided in this work should be at least qualitatively correct. For the *J* = 1 case, the protocol employed in this work is not suitable for a direct calculation of the experimentally observed rotational constants *A*_**ν**_, *B*_**ν**_, and *C*_**ν**_ because only the interaction between pseudorotational and rotational motions is calculated and taken into account (vibration–rotation interactions involving the other 3*K* – 5 internal motions cannot be safely neglected). Nevertheless, *differences* of the ν-specific rotational constant values reported in Tables 1–3 could be a reasonable estimation of the experimental ones, at least for the lowest eigenvalues (in previous contributions,^76,77^ the original Meyer’s implementation of the 1D flexible model was employed to reproduce the experimental variation of rotational constants associated with different values of the index ν).

*Limitations:* The dynamical contribution of all the other 3*K* – 5 internal degrees of freedom is neglected, and the full dimensional NSE is reduced to a 1D-NSE. Furthermore, the rovibrational problem is formulated and solved only in the *J* = 0 and *J* = 1 cases, while formulations for the *J* > 1 cases (available in literature) are not discussed.

*Possible Improvements:* A proper inclusion of the coupling between the pseudorotational motion and the other internal degrees of freedom would be useful, especially for the computational characterization of pseudorotational pathways with very small potential energy barriers (less than 100 cm^–1^) or for an accurate determination of higher-energy eigenvalues of *Ĥ*_vib_^0^. Different strategies were proposed and implemented, such as the formulation of an effective 1D nuclear Hamiltonian (BO-like separation between the pseudorotational motion and the other internal degrees of freedom)^24−26^ or the formulation of a higher-dimensional nuclear Hamiltonian in curvilinear coordinates (2D,^19^ 3D,^28^ or even full dimensional^30^ formulations). With regard to the *J* = 1 case, a complete calculation of the vibration–rotation interaction (taking into account *all* the internal motions) would allow the calculation of experimentally observed rotational constants *A*_**ν**_, *B*_**ν**_, and *C*_**ν**_. An extension devoted to the formulation of the nuclear Hamiltonian (and to the solution of the nuclear problem) for higher values of *J* (*J* > 1) would be also useful.^27,29^

## 6 Conclusions

In this work, the implementation of an approach suitable for the calculation and the assignment of ν-specific rotational constants *A*_ν_, *B*_ν_, and *C*_ν_ is described and the solutions to the 1D-NSEs (for *J* = 0 and *J* = 1) associated with the pseudorotational motion of 1,2-dioxolane, 1,2-oxathiolane, and 3-chloro-1,2-dithiolane are reported as test cases. The implementation proposed is suitable for a computational characterization of the pseudorotational motion in 5-term ring systems; in particular, a procedure to perform a relaxed scan along the pseudorotation angle ϕ (defined according to the original definition proposed by Cremer and Pople) without the explicit employment of the analytical gradient was devised, implemented, and applied.

From another point of view, this work is an initial assessment of usefulness and advantages of CPCs for the formulation of the NSE. The usefulness of CPCs and RDCs for the construction of *n*D-PESs (or to perform conformational studies) is well-known,^33,78−81^ and new contributions concerning these coordinate systems and their generalization appeared in the literature in the last two years;^81,82^ therefore, in the opinion of the authors the development and the assessment of computational tools aiming at the employment of these coordinates in a quantum mechanical description of the nuclear motions is worthwhile.

Limitations and possible improvements of the 1D model discussed in this contribution are mentioned.

## Supporting Information Available

Relaxed scan along the Cremer–Pople pseudorotation angle ϕ; construction and implementation of the Hamiltonian operators *Ĥ*_vib_^0^ and *Ĥ*_vibrot_^1^; 1D-NSE as a Sturm–Liouville problem; choice of the reference system: the case of 1,2-dioxolane (PDF)
