# Supplementary material for: Interplay of Rotational and Pseudorotational Motions in Flexible Cyclic Molecules
Source: J Phys Chem A. 2021 May 11;125(19):4098–113. doi: 10.1021/acs.jpca.1c01472 (PMC8279653; doi:10.1021/acs.jpca.1c01472)
Supplement: Supplementary file 1 — jp1c01472_si_001.pdf [file jp1c01472_si_001.pdf]

SUPPLEMENTARY MATERIAL for: Interplay of rotational and pseudorotational motions in flexible cyclic molecules

Lorenzo Paoloni[1,*] and Assimo Maris[2]

[1]*Dipartimento di Fisica e Astronomia, Università di Padova, via Marzolo 8, I-35131 Padova, Italy.*
[2]*Dipartimento di Chimica G. Ciamician, Università di Bologna, via Selmi 2, I-40126 Bologna, Italy.*
[*]*Corresponding author: Lorenzo Paoloni, lorenzo.paoloni@unipd.it*

Contents

1 Relaxed scan along the Cremer-Pople pseudorotation angle ϕ SI 1
1.1 Choice of constraints and description of the algorithm . . . . SI 1
1.2 Reduction of a 2D-PES: employment of an analytical formulation to express the optimal q value . . . . SI 3

2 Construction and implementation of the hamiltonian operators $\hat{H}^0_{vib}$ and $\hat{H}^1_{vibrot}$ SI 5
2.1 Elements needed for the numerical calculation of the kinetic energy operator . . . . SI 5
2.2 Matrix formulations of the nuclear hamiltonians . . . . SI 7
2.2.1 Formulation of $\hat{H}^0_{vib}$ . . . . SI 7
2.2.2 Formulation of $\hat{H}^1_{vibrot}$ . . . . SI 14

3 The 1D-NSE as a Sturm-Liouville problem SI 22
3.1 First case: $g^{\phi\phi}(\phi) = const$ and $P(\phi) = const$ . . . . SI 22
3.2 Second case: $g^{\phi\phi}(\phi) = const$ and $P(\phi) = f(\phi)$ . . . . SI 25

4 Choice of the reference system: the case of 1,2-dioxolane SI 28

1 Relaxed scan along the Cremer-Pople pseudorotation angle ϕ

1.1 Choice of constraints and description of the algorithm

In what follows, a detailed description of the algorithm employed for the construction of the one-dimensional cut of the potential energy surface (1D-PES) along the pseudorotation angle (ϕ) is provided. Each point was calculated independently through a ϕ constrained optimization. The molecular structure was specified in each input employing cartesian and Primitive Internal Coordinates (PICs) in the same Z-matrix. Cartesian coordinates (in the framework specified by eqs. 1 and 5 in the main text of the article) were employed to specify the positions of the five atoms directly involved in the 5-term ring system, while PICs were used to specify the positions of all the other atoms. In particular, the z values of these five atoms correspond to a specific couple of Ring Puckering Coordinates (the puckering amplitude q and the pseudorotation angle ϕ). The algorithm employed to automatically select simple constraints to freeze the value of ϕ while all the other nuclear degrees of freedom (including q) are optimized is introduced below.

The starting point is the formulation of the pseudorotation angle ϕ for a 5-term ring system:[SI1–SI3]

$$\phi = \begin{cases} \arctan\left[-\frac{\sum_{j=1}^{5} z_j \sin\left(\frac{4\pi(j-1)}{5}\right)}{\sum_{j=1}^{5} z_j \cos\left(\frac{4\pi(j-1)}{5}\right)}\right] & \text{if } \cos\phi > 0 \\ \arctan\left[-\frac{\sum_{j=1}^{5} z_j \sin\left(\frac{4\pi(j-1)}{5}\right)}{\sum_{j=1}^{5} z_j \cos\left(\frac{4\pi(j-1)}{5}\right)}\right] + \pi & \text{if } \cos\phi < 0 \\ \operatorname{sgn}[\sin(\phi)]\frac{\pi}{2} & \text{if } \cos\phi = 0 \end{cases} \tag{SI-1}$$

To perform a relaxed scan along ϕ the following condition must be fulfilled:

$$\arctan\left[-\frac{\sum_{j=1}^{5} z_j \sin\left(\frac{4\pi(j-1)}{5}\right)}{\sum_{j=1}^{5} z_j \cos\left(\frac{4\pi(j-1)}{5}\right)}\right] = const. \tag{SI-2}$$

The condition given in eq. SI-2 can be reduced to:

$$\frac{\sum_{j=1}^{5} z_j \sin\left(\frac{4\pi(j-1)}{5}\right)}{\sum_{j=1}^{5} z_j \cos\left(\frac{4\pi(j-1)}{5}\right)} = const. \tag{SI-3}$$

In the last version of the GAUSSIAN suite of programs[SI4] user-defined constraints can be employed by means of suitable definitions of the so-called Generalized Internal Coordinates (GICs). It can be shown that this condition can be fulfilled if the four constraints $\frac{z_1}{z_2} = k_1 = const$, $\frac{z_2}{z_3} = k_2 = const$, $\frac{z_3}{z_4} = k_3 = const$ and

$\frac{z_4}{z_5} = k_4 = const$ are imposed. Indeed $z_1 = k_1 z_2 = k_1 k_2 z_3 = k_1 k_2 k_3 z_4 = k_1 k_2 k_3 k_4 z_5$ and similarly $z_2 = k_2 k_3 k_4 z_5$, $z_3 = k_3 k_4 z_5$, $z_4 = k_4 z_5$ so that:

$$\frac{z_1 \sin\left(\frac{4\pi(0)}{5}\right) + z_2 \sin\left(\frac{4\pi}{5}\right) + z_3 \sin\left(\frac{8\pi}{5}\right) + z_4 \sin\left(\frac{12\pi}{5}\right) + z_5 \sin\left(\frac{16\pi}{5}\right)}{z_1 \cos\left(\frac{4\pi(0)}{5}\right) + z_2 \cos\left(\frac{4\pi}{5}\right) + z_3 \cos\left(\frac{8\pi}{5}\right) + z_4 \cos\left(\frac{12\pi}{5}\right) + z_5 \cos\left(\frac{16\pi}{5}\right)} =$$
$$\frac{k_1 k_2 k_3 k_4 z_5 (0) + k_2 k_3 k_4 z_5 \sin\left(\frac{4\pi}{5}\right) + k_3 k_4 z_5 \sin\left(\frac{8\pi}{5}\right) + k_4 z_5 \sin\left(\frac{12\pi}{5}\right) + z_5 \sin\left(\frac{16\pi}{5}\right)}{k_1 k_2 k_3 k_4 z_5 (1) + k_2 k_3 k_4 z_5 \cos\left(\frac{4\pi}{5}\right) + k_3 k_4 z_5 \cos\left(\frac{8\pi}{5}\right) + k_4 z_5 \cos\left(\frac{12\pi}{5}\right) + z_5 \cos\left(\frac{16\pi}{5}\right)} =$$
$$\frac{k_2 k_3 k_4 \sin\left(\frac{4\pi}{5}\right) + k_3 k_4 \sin\left(\frac{8\pi}{5}\right) + k_4 \sin\left(\frac{12\pi}{5}\right) + \sin\left(\frac{16\pi}{5}\right)}{k_1 k_2 k_3 k_4 + k_2 k_3 k_4 \cos\left(\frac{4\pi}{5}\right) + k_3 k_4 \cos\left(\frac{8\pi}{5}\right) + k_4 \cos\left(\frac{12\pi}{5}\right) + \cos\left(\frac{16\pi}{5}\right)}. \tag{SI-4}$$

The last term of eq. SI-4 is constant.*

The strategy proposed above is not suitable when one of the five z_j values is equal to 0 (because the corresponding constraint k is not defined due to a division by zero).† In principle, to overcome this difficulty another constraint can be defined ($\frac{z_5}{z_1} = k_5$) and the constraint k whose denominator corresponds to the smallest absolute value of the five $|z|$ values can be removed while the other four constraints are imposed (e.g. if $z_3 = 0$, the constraints k_1, k_3, k_4 and k_5 are employed and the constraint k_2 is neglected). In practice, this solution leads to a situation in which one of the four constraints k can assume an exceedingly small value (in terms of absolute values) with respect to the other three constraints (e.g. if z_3 is not equal but close to zero, then $|k_3| << |k_1|$, $|k_3| << |k_4|$ and $|k_3| << |k_5|$): in order to carry out the corresponding constrained optimization in an effective manner, values of the constraints k with the same order of magnitude are desirable. The algorithm employed in this work to select the user-defined constraints inserted in the GAUSSIAN input files (used for the constrained optimizations in the full range of values $[0,2\pi]$ of the pseudorotation angle ϕ) is the following one:

1. among the five atoms which are involved in the central 5-term ring with z_j values z_1, z_2, z_3, z_4 and z_5, find the one with the smallest absolute value;
2. define three constraints k as ratios which involve the four z_j values not selected in the previous step (e.g. if the absolute value of z_3 is the smallest one, the constraints $k_1 = \frac{z_1}{z_2}$, $k_4 = \frac{z_4}{z_5}$ and $k_5 = \frac{z_5}{z_1}$ are defined);
3. introduce the constraint $z_1 + z_2 + z_3 + z_4 + z_5 = 0$ (i.e. the sum of the five z values must be equal to zero). This condition is employed as a constraint for the z_j value with the smallest absolute value.

*Because $k_1 = const$, $k_2 = const$, $k_3 = const$ and $k_4 = const$.

†For the case of z_1 the situation is different, because this coordinate is not involved in the denominators of k_1, k_2, k_3 and k_4.

With the algorithm described above, four simple constraints (three ratios and one summation) are added in each input file through the definition of four GICs, which are frozen in order to set the value of the pseudorotation angle ϕ (which is constant during the constrained optimization) while the other nuclear degrees of freedom are optimized.

1.2 Reduction of a 2D-PES: employment of an analytical formulation to express the optimal q value

With regards to the three molecular systems investigated in this work, the 1D-PES along the pseudorotation angle can be obtained employing a different strategy. In ref. SI3 suitable analytical formulations of the 2D-PESs were reported and discussed: these analytical formulations can be employed to extract an optimal value of q, i.e. to reduce the 2D-PES $V(q,\phi)$ to a 1D-PES $V(\phi) = V(q_{opt}(\phi),\phi)$. This solution is not convenient in the most general case, because the direct calculation of a 1D-PES (which is possible with the algorithm proposed in the previous subsection) is less demanding than the calculation of a 2D-PES, but when the analytical formulation of the 2D-PES is already available from a previous study the most demanding step of the computational procedure is skipped and the solution mentioned in this subsection can be useful. In what follows, the procedure is detailed for the case of 1,2-dioxolane.

Employing the following analytical formulation for the 2D-PES associated to the ring puckering of 1,2-dioxolane (see table 4 of ref. SI3):

$$V(q,\phi) = a + b_1 q^2 + b_2 q^4 + b_{12} q^2 \cos(2\phi) + b_{22} q^4 \cos(2\phi); \tag{SI-5}$$

the minimisation of $V(q,\phi)$ with respect to q can be performed analytically, applying the condition $\frac{\partial V(q,\phi)}{\partial q} = 0$:

$$\frac{\partial V(q,\phi)}{\partial q} = 2b_1 q + 4b_2 q^3 + 2b_{12} q \cos(2\phi) + 4b_{22} q^3 \cos(2\phi) = 0. \tag{SI-6}$$

Eq. SI-6 can be rewritten as follows:

$$\begin{aligned} &2b_1 q + 4b_2 q^3 + 2b_{12} q \cos(2\phi) + 4b_{22} q^3 \cos(2\phi) = \\ &q\left[2b_1 + 4b_2 q^2 + 2b_{12} \cos(2\phi) + 4b_{22} q^2 \cos(2\phi)\right] = 0; \end{aligned} \tag{SI-7}$$

the trivial solution of eq. SI-7 ($q = 0$) corresponds to the planar conformation. The other solutions can be easily found:

$$2b_1 + 4b_2 q^2 + 2b_{12} \cos(2\phi) + 4b_{22} q^2 \cos(2\phi) = 0; \tag{SI-8}$$

which leads to the following formulation of q_{opt}:

$$q_{opt}^2 = -\frac{1}{2}\frac{b_1 + b_{12}\cos(2\phi)}{b_2 + b_{22}\cos(2\phi)}; \tag{SI-9}$$

Eq. SI-9 can be employed to obtain $V(\phi)$ as $V(q_{opt}(\phi),\phi)$: in this manner, the 1D-PES can be constructed without employing the procedure proposed in the previous subsection. However, to solve the rovibrational problem a suitable formulation of $\hat{H}^0_{vib}$ and $\hat{H}^1_{vibrot}$ is needed. This means that analytical formulations of nuclear coordinates as functions of q and ϕ should be available, i.e. relationships of the kind $S_{i\alpha}(q,\phi)$. If the relationships $S_{i\alpha}(q,\phi)$ are available, the structures corresponding to optimal values of q_{opt} can be easily obtained employing eq. SI-9, because $S_{i\alpha}(\phi) = S_{i\alpha}(q_{opt}(\phi),\phi)$.

2 Construction and implementation of the hamiltonian operators $\hat{H}^0_{vib}$ and $\hat{H}^1_{vibrot}$

To ensure the reproducibility of the results provided in this work, the numerical procedures employed for the construction of the nuclear hamiltonian matrices $\hat{H}^0_{vib}$ and $\hat{H}^1_{vibrot}$ must be detailed. As mentioned in the main text, the formulation of $\hat{H}^0_{vib}$ and $\hat{H}^1_{vibrot}$ proposed by Meyer[SI5] was employed. However, two differences between the original implementation of Meyer and the one proposed in this work should be mentioned: (i) the implementation proposed by Meyer was devoted to the formulation of $\hat{H}^0_{vib}$ and $\hat{H}^1_{vibrot}$ and to the solution of the associated eigenvalue problems, but nowadays it would require an additional code devoted to the extraction of structures and energies from the output of a series of constrained optimizations: this last part is already included in the implementation proposed in this work; (ii) the choice of the molecule-fixed axes suggested in ref. SI5 is different from the ones employed in this work.

2.1 Elements needed for the numerical calculation of the kinetic energy operator

Employing the computational procedure proposed in the previous section, the pseudorotation of a saturated 5-term ring system can be described with a single independent variable: the pseudorotation angle ϕ. This means that:

$$\begin{array}{llll} E = E(\phi) & \text{and} & & r_{i\alpha} = r_{i\alpha}(\phi) \\ \text{with} & i = 1,\ldots,K & \text{and} & \alpha = 1,2,3. \end{array} \tag{SI-10}$$

In SI-10, K is the total number of atoms of the molecular system and $r_{i\alpha}$ is a single cartesian coordinate of one of the nuclei. To avoid any ambiguity, the reference system of the cartesian framework must be specified. More specifically, the origin of the cartesian framework employed in the solution of the rovibrational problem coincides with the center of mass of the entire molecular system.* An explicit analytical formulation of the functions $E(\phi)$ and $r_{i\alpha}(\phi)$ is not available, but the value of $E(\phi_j)$ and $r_{i\alpha}(\phi_j)$ at a specific point ϕ_j can be calculated through a constrained optimization where $\phi = \phi_j$ and all the other coordinates are optimized. Therefore, $E(\phi_j)$ and $r_{i\alpha}(\phi_j)$ can be obtained from the output of a single

*In the main text and in the previous section of the SI, conformations of the 5-term saturated ring systems are specified with the two RPCs q and ϕ: to define q and ϕ, the cartesian framework specified in eqs. 1-5 of the main text must be employed. Eq. 1 of the main text specify an origin of the cartesian framework which is equal to the geometrical center of the nuclei directly involved in the structure of the 5-term ring system: this prescription cannot be applied for the formulation of the rovibrational hamiltonian proposed in this section. Therefore, in eq. SI-10 and for all the other occurrences of $r_{i\alpha}$ in this section and in the next ones, the origin of the cartesian framework is equal to the center of mass of the entire molecular system and *not* to the geometrical center specified in eq. 1 of the main text.

constrained optimization. The classical kinetic energy matrix at $\phi = \phi_j$ can be calculated as follows:

$$\begin{bmatrix} g_{11} & g_{12} & g_{13} & g_{1\phi} \\ g_{21} & g_{22} & g_{23} & g_{2\phi} \\ g_{31} & g_{32} & g_{33} & g_{3\phi} \\ g_{\phi 1} & g_{\phi 2} & g_{\phi 3} & g_{\phi\phi} \end{bmatrix}_{\phi=\phi_j} =$$

$$\begin{bmatrix} \sum_i^K [m_i \sum_{\beta,\beta'}^3 (\varepsilon_{i1\beta'} r_{i\beta'} \varepsilon_{i1\beta} r_{i\beta})] & \sum_i^K [m_i \sum_{\beta,\beta'}^3 (\varepsilon_{i1\beta'} r_{i\beta'} \varepsilon_{i2\beta} r_{i\beta})] & \sum_i^K [m_i \sum_{\beta,\beta'}^3 (\varepsilon_{i1\beta'} r_{i\beta'} \varepsilon_{i3\beta} r_{i\beta})] & \sum_i^K [m_i \sum_{\alpha,\beta'}^3 (\varepsilon_{i1\beta'} r_{i\beta'} \frac{dr_{i\alpha}}{d\phi})] \\ \sum_i^K [m_i \sum_{\beta,\beta'}^3 (\varepsilon_{i2\beta'} r_{i\beta'} \varepsilon_{i1\beta} r_{i\beta})] & \sum_i^K [m_i \sum_{\beta,\beta'}^3 (\varepsilon_{i2\beta'} r_{i\beta'} \varepsilon_{i2\beta} r_{i\beta})] & \sum_i^K [m_i \sum_{\beta,\beta'}^3 (\varepsilon_{i2\beta'} r_{i\beta'} \varepsilon_{i3\beta} r_{i\beta})] & \sum_i^K [m_i \sum_{\alpha,\beta'}^3 (\varepsilon_{i2\beta'} r_{i\beta'} \frac{dr_{i\alpha}}{d\phi})] \\ \sum_i^K [m_i \sum_{\beta,\beta'}^3 (\varepsilon_{i3\beta'} r_{i\beta'} \varepsilon_{i1\beta} r_{i\beta})] & \sum_i^K [m_i \sum_{\beta,\beta'}^3 (\varepsilon_{i3\beta'} r_{i\beta'} \varepsilon_{i2\beta} r_{i\beta})] & \sum_i^K [m_i \sum_{\beta,\beta'}^3 (\varepsilon_{i3\beta'} r_{i\beta'} \varepsilon_{i3\beta} r_{i\beta})] & \sum_i^K [m_i \sum_{\alpha,\beta'}^3 (\varepsilon_{i3\beta'} r_{i\beta'} \frac{dr_{i\alpha}}{d\phi})] \\ \sum_i^K [m_i \sum_{\alpha,\beta}^3 (\frac{dr_{i\alpha}}{d\phi} \varepsilon_{i1\beta} r_{i\beta})] & \sum_i^K [m_i \sum_{\alpha,\beta}^3 (\frac{dr_{i\alpha}}{d\phi} \varepsilon_{i2\beta} r_{i\beta})] & \sum_i^K [m_i \sum_{\alpha,\beta}^3 (\frac{dr_{i\alpha}}{d\phi} \varepsilon_{i3\beta} r_{i\beta})] & \sum_i^K [m_i \sum_{\alpha}^3 (\frac{dr_{i\alpha}}{d\phi})^2] \end{bmatrix}_{\phi=\phi_j} ; \quad \text{(SI-11)}$$

where ε_{ijk} is the total antisymmetric unit tensor (or Levi-Civita symbol) and m_i is the mass of the i-th nucleus. The first derivative $\frac{dr_{i\alpha}}{d\phi}$ can be easily calculated numerically. The kinetic energy matrix is the inverse of its classical analogue:

$$\begin{bmatrix} g^{11} & g^{12} & g^{13} & g^{1\phi} \\ g^{21} & g^{22} & g^{23} & g^{2\phi} \\ g^{31} & g^{32} & g^{33} & g^{3\phi} \\ g^{\phi 1} & g^{\phi 2} & g^{\phi 3} & g^{\phi\phi} \end{bmatrix}_{\phi=\phi_j} = \begin{bmatrix} g_{11} & g_{12} & g_{13} & g_{1\phi} \\ g_{21} & g_{22} & g_{23} & g_{2\phi} \\ g_{31} & g_{32} & g_{33} & g_{3\phi} \\ g_{\phi 1} & g_{\phi 2} & g_{\phi 3} & g_{\phi\phi} \end{bmatrix}_{\phi=\phi_j}^{-1} . \quad \text{(SI-12)}$$

For the calculation of the pseudopotential $\hat{V}'(\phi)$ the determinant of the classical kinetic energy matrix is needed:

$$g(\phi) = \begin{vmatrix} g_{11} & g_{12} & g_{13} & g_{1\phi} \\ g_{21} & g_{22} & g_{23} & g_{2\phi} \\ g_{31} & g_{32} & g_{33} & g_{3\phi} \\ g_{\phi 1} & g_{\phi 2} & g_{\phi 3} & g_{\phi\phi} \end{vmatrix}_{\phi=\phi_j} . \quad \text{(SI-13)}$$

For each value of the pseudorotation angle $\phi = \phi_j$, the quantities needed for the construction of $\hat{T}$ are g (eq. SI-13) and the elements of the kinetic energy matrix (left hand side of eq. SI-12). In the case $J = 0$ (pure pseudorotational motion), only the quantities g and $g^{\phi\phi}$ are needed.

In principle, the calculation of the quantities mentioned above requires the knowledge of the molecular structure (after the constrained optimization) at each point ϕ_j. In practice, the knowledge of these quantities for a limited number of values of ϕ can be employed to interpolate the same quantities at other values of ϕ. This is important for at least two reasons: (i) with the method employed in this work, the number of solutions (i.e. number of eigenstates) to the eigenvalue problem associated to the nuclear hamiltonian matrix is equal to the number of mesh points[†] and (ii) the number of optimized structures which are needed for an

[†]The mesh points are the number of values of the pseudorotation angle ϕ employed for the construction of $\hat{V}$ and $\hat{T}$ (and therefore for the construction of $\hat{H}^0_{vib}$).

accurate numerical calculation of $\hat{T}$ can be reduced.[‡] Another advantage of this procedure is the smaller number of matrix inversions required (see eq. SI-12).

In this work, constrained optimizations (at fixed values of ϕ) were carried out at 360 values of ϕ[§] (one constrained optimization for each degree, i.e. for values of $\phi = 1°, 2°, \ldots$), but the nuclear hamiltonian matrices $\hat{H}^0_{vib}$ were constructed and diagonalized on a grid of 1800 mesh points: the 360 values of each of the elements of the kinetic energy matrix, of $g(\phi)$ and $E(\phi)$ were used to interpolate all the other mesh points with a suitable Fourier series.

All the terms of the Fourier series up to an order chosen by the operator were employed to interpolate additional points of all the quantities of interest in the case of 3-chloro-1,2-dithiolane.[¶] In the case of 1,2-dioxolane and 1,2-oxathiolane, symmetry was employed to select the terms of the Fourier series employed to interpolate the points of g, $g^{\phi\phi}$ and E (in the case of E, the symmetries discussed in ref. SI3 were employed; with regards to the calculations performed for this work, those symmetries were employed also for g and $g^{\phi\phi}$). All the other elements of the kinetic energy matrix were interpolated without assuming the presence of a symmetry different from $f(\phi) = f(\phi + 2\pi)$.[‖]

2.2 Matrix formulations of the nuclear hamiltonians

2.2.1 Formulation of $\hat{H}^0_{vib}$

The pseudorotational motion was described by the pseudorotation angle ϕ which assumes values in the interval $[-\pi, +\pi]$: following Meyer,[SI5,SI6] $2M + 1$ equally spaced mesh points were used. More specifically, the following definition was adopted for a specific mesh point ϕ_j:

$$\phi_j = \frac{j2\pi}{2M+1} \qquad \text{with} \qquad j = 0, \pm 1, \ldots, \pm M. \tag{SI-14}$$

In order to formulate the hamiltonians $\hat{H}^0_{vib}$ and $\hat{H}^1_{vibrot}$, a matrix formulation of the differential operator $\frac{d}{d\phi}$ is needed:

$$f'(\phi_{j'}) = \sum_{j=-M}^{M} D'_{j'j} f(\phi_j). \tag{SI-15}$$

With regards to the pseudorotation of 5-term ring systems, the function $f(\phi)$ is periodic, i.e. $f(\phi) = f(\phi + 2\pi)$; therefore, $f(\phi)$ can be approximated with a Fourier series of order M:

‡It must be pointed out that this reduction should not affect the final result, i.e. at the end of the procedure a reliable interpolation must be possible.

§For each of the molecular systems studied.

¶With regards to the results provided in this work, the first 40 terms of the Fourier series were employed (41, if the known term is counted), i.e. up to the coefficients of the 20-th harmonic.

‖Also in the cases of 1,2-dioxolane and 1,2-oxathiolane the first 40 terms of the Fourier series were employed.

$$f(\phi) = \frac{1}{2}a_0 + \sum_{k=1}^{M} \left[a_k \cos(k\phi) + b_k \sin(k\phi)\right]; \tag{SI-16}$$

where the $2M+1$ expansion coefficients can be related to the values $f(\phi_j)$ (i.e. to the values of f corresponding to the $2M+1$ mesh points ϕ_j) with the following relationships:*

$$a_k = \frac{2}{2M+1} \sum_{j=-M}^{M} \left[f(\phi_j)\cos(k\phi_j)\right]; \tag{SI-17}$$

$$b_k = \frac{2}{2M+1} \sum_{j=-M}^{M} \left[f(\phi_j)\sin(k\phi_j)\right]. \tag{SI-18}$$

The analytical formulation of $f'(\phi)$ can be easily obtained from the first derivative of eq. SI-16: employing eqs. SI-17 and SI-18, an expression of $f'(\phi)$ which exhibits a linear dependence from the values $f(\phi_j)$ can be found (i.e. the coefficients of this expression are the elements $D'_{j'j}$ of eq. SI-15):

$$\begin{aligned} f'(\phi) &= \frac{2}{2M+1}\left\{ \sum_{k=1}^{M}\sum_{j=-M}^{+M} \left[k f(\phi_j)\sin(k\phi_j)\cos(k\phi)\right] - \sum_{k=1}^{M}\sum_{j=-M}^{+M} \left[k f(\phi_j)\cos(k\phi_j)\sin(k\phi)\right] \right\} \\ &= \sum_{j=-M}^{M} \left\{ \frac{2}{2M+1} f(\phi_j) \sum_{k=1}^{M} \left[k\Big(\sin(k\phi_j)\cos(k\phi) - \cos(k\phi_j)\sin(k\phi)\Big)\right] \right\} \\ &= \sum_{j=-M}^{M} \left\{ \frac{2}{2M+1} \sum_{k=1}^{M} \left[k \sin\big(k(\phi_j - \phi)\big)\right] \right\} f(\phi_j). \end{aligned} \tag{SI-19}$$

If the continuous variable ϕ is substituted by the discrete variable $\phi_{j'}$, the final result of eq. SI-19 can be written as follows:

$$f'(\phi_{j'}) = \sum_{j=-M}^{M} \overbrace{\left\{ \frac{2}{2M+1} \sum_{k=1}^{M} \left[k \sin\big(k(\phi_j - \phi_{j'})\big)\right] \right\}}^{D'_{j'j}} f(\phi_j); \tag{SI-20}$$

where the summation can be carried out. The result is the following one:[SI5,SI6]

$$D'_{j'j} = \frac{2}{2M+1} \sum_{k=1}^{M} \left\{k \sin\left[k(\phi_j - \phi_{j'})\right]\right\} = \begin{cases} (-1)^{j'-j}\left[\frac{1}{2\pi \sin\left(\frac{j'-j}{2M+1}\right)}\right] & \text{for} \quad j' \neq j \\ 0 \quad \text{for} \quad j' = j \end{cases} \tag{SI-21}$$

*Eq. SI-17 and SI-18 are the discrete versions of $a_k = \frac{2}{T}\int_{-\frac{T}{2}}^{\frac{T}{2}} f(\phi)\cos(\frac{2\pi}{T}k\phi)d\phi$ and $b_k = \frac{2}{T}\int_{-\frac{T}{2}}^{\frac{T}{2}} f(\phi)\sin(\frac{2\pi}{T}k\phi)d\phi$ (where T is the period of the function), respectively.

For a complete formulation of $\hat{H}^0_{vib}$, a suitable formulation of the ϕ-dependent pseudopotential term V' is needed (see eq. 3.6 of ref. SI5):

$$V'(\phi) = \frac{\hbar^2}{2}\left[g^{\phi\phi}\left(\frac{1}{4}\frac{d\ln g(\phi)}{d\phi}\right)^2 + \frac{d\left(\frac{1}{4}g^{\phi\phi}\frac{d\ln g(\phi)}{d\phi}\right)}{d\phi}\right]. \tag{SI-22}$$

Two of the quantities on the RHS of eq. SI-22 ($g^{\phi\phi}$ and g) were discussed previously (see the previous subsection). The derivatives with respect to the independent variable ϕ were calculated numerically.†

The solution of the nuclear problem is achieved by means of a pseudospectral or DVR method, i.e. the matrix formulation of $\hat{H}^0_{vib}$ is provided in terms of a pseudospectral basis. The basis is the set of M-th elements of Dirichlet kernels centred at each of the equally spaced $2M+1$ mesh points ϕ_j. The mathematical formulation of the M-th element of the Dirichlet kernel centred on the mesh point ϕ_j is the following one (see eq. 3.10 of ref. SI5 or eq. 4.8 of ref. SI6):

$$D^j_M(\phi) = \frac{1}{2\pi}\frac{\sin[(M+\frac{1}{2})(\phi-\phi_j)]}{\sin[\frac{1}{2}(\phi-\phi_j)]}. \tag{SI-23}$$

Elements pertaining to the basis proposed in eq. SI-23 are orthogonal (i.e. $\int_{-\pi}^{+\pi} D^j_M D^{j'}_M d\phi = 0$ if $j \neq j'$),‡ but not orthonormal. The spacing between two adjacent mesh points can be labeled and written as follows:

$$\Delta\phi = \frac{2\pi}{2M+1}. \tag{SI-24}$$

Employing $\sqrt{\Delta\phi}$ as a multiplicative constant factor for the function $D^j_M(\phi)$, a set of orthonormal functions is obtained.§

When $\phi = \phi_{j'}$ (where $\phi_{j'} = \frac{2\pi j'}{2M+1}$, see eq. SI-14), the values of the function proposed in eq. SI-23 are the following ones:¶

$$D^j_M(\phi_{j'}) = \frac{1}{2\pi}\frac{\sin[(M+\frac{1}{2})(\phi_{j'}-\phi_j)]}{\sin[\frac{1}{2}(\phi_{j'}-\phi_j)]} = \begin{cases} \frac{1}{\Delta\phi} & \text{if} \quad j'=j \\ 0 & \text{if} \quad j' \neq j \end{cases}. \tag{SI-25}$$

The analytical formulation proposed in eq. SI-23 is equivalent to the following summations (see eqs. 4.6 and 4.7 of ref. SI6):

† with regards to the quantity $\ln g(\phi)$, the second derivative is needed: therefore, numerical inaccuracies concerning the value of $\ln g(\phi)$ should be minimised.

‡ The functions D^j_M are periodic ($D^j_M(\phi) = D^j_M(\phi+2\pi)$), with a period of 2π.

§ i.e. $\int_{-\pi}^{+\pi}\sqrt{\Delta\phi}D^j_M\sqrt{\Delta\phi}D^{j'}_M d\phi = \int_{-\pi}^{+\pi}\Delta\phi D^j_M D^{j'}_M d\phi = \delta_{j'j}$, where $\delta_{j'j}$ is the Kronecker delta.

¶ For the case $j'=j$, the result can be easily obtained employing the L'Hôpital's rule for the calculation of $\lim_{\phi\to\phi_j} D^j_M(\phi)$

$$\frac{1}{2\pi}\frac{\sin[(M+\frac{1}{2})(\phi-\phi_j)]}{\sin[\frac{1}{2}(\phi-\phi_j)]}=\frac{1}{2\pi}\sum_{k=-M}^{M}\left[e^{ik(\phi-\phi_j)}\right]=\frac{1}{2\pi}\left\{1+2\sum_{k=1}^{M}\cos[k(\phi-\phi_j)]\right\}. \tag{SI-26}$$

In fig. SI 1, seven elements of the basis set D^j_{180} are depicted (multiplied for the constant value $\Delta\phi$).

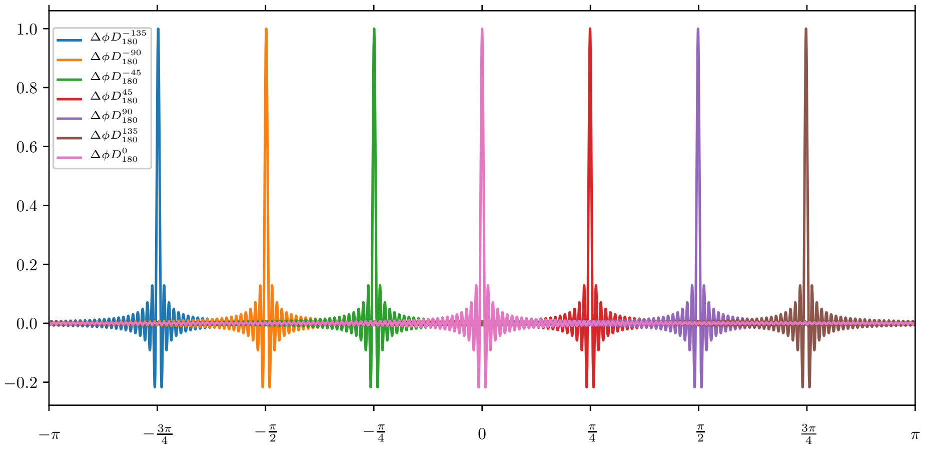

Figure SI 1: Graphical representation of $\Delta\phi D^j_{180}(\phi)$; j values are specified in the legend.

From the mathematical point of view, the usefulness of a function $D^j_M(\phi)$ is related to its convolution with a generic function $f(\phi)$ of period 2π, which is equal to the Fourier series approximation (at M-th order) of $f(\phi)$ (see the exponential formulation of D^j_M provided in eq. SI-26): therefore, the employment of the Dirichlet kernels is a transparent way to introduce the approximation of eq. SI-16 to $f(\phi)$. Moreover, the derivative of $D^j_M(\phi)$ can be easily calculated from the last term of eq. SI-26:

$$\left.\frac{dD^j_M}{d\phi}\right|_{\phi_{j'}}=-\frac{1}{\pi}\sum_{k=1}^{M}\left\{k\sin\left[k(\phi_{j'}-\phi_j)\right]\right\}=\frac{1}{\pi}\sum_{k=1}^{M}\left\{k\sin\left[k(\phi_j-\phi_{j'})\right]\right\}=\frac{D'_{j'j}}{\Delta\phi}; \tag{SI-27}$$

where $D'_{j'j}$ is defined in eq. SI-21. Therefore, the following relationship holds:

$$\left.\frac{dD^j_M}{d\phi}\right|_{\phi_{j'}}\Delta\phi=D'_{j'j}. \tag{SI-28}$$

A projection operator can be defined as follows:[‖]

[‖]Eq. SI-29 can be rewritten as follows: $\hat{O}_v=\sum_{j=-M}^{+M}\left[\sqrt{\Delta\phi}\left|D^j_M\right\rangle\sqrt{\Delta\phi}\left\langle\phi_j\right|\right]$. If $\left|B^j_M\right\rangle=\sqrt{\Delta\phi}\left|D^j_M\right\rangle$, the projection operator will become: $\hat{O}_v=\sum_{j=-M}^{+M}\left[\left|B^j_M\right\rangle\sqrt{\Delta\phi}\left\langle\phi_j\right|\right]$.

$$\hat{O}_v = \sum_{j=-M}^{+M} \left[\left| D_M^j \right\rangle \Delta\phi \left\langle \phi_j \right| \right], \tag{SI-29}$$

where the Dirac notation is introduced. The fundamental property $\hat{O}_v^2 = \hat{O}_v$ can be easily verified.**

Employing eq. SI-29, a formulation of $\hat{H}^0_{vib}$ and of wavefunctions in terms of the pseudospectral basis functions D_M^j is possible. With an orthonormal basis such as $\left| B_M^j \right\rangle = \sqrt{\Delta\phi} \left| D_M^j \right\rangle$, the matrix formulation of $\hat{H}^0_{vib}$ can be written as follows:††

$$\hat{H}^0_{vib_{j'j}} = -\frac{\hbar^2}{2} \left\langle B_M^{j'} \right| \frac{\partial}{\partial\phi} \left[g^{\phi\phi}(\phi) \frac{\partial}{\partial\phi} \right] \left| B_M^j \right\rangle + \left\langle B_M^{j'} \right| \hat{V}'(\phi) \left| B_M^j \right\rangle + \left\langle B_M^{j'} \right| \hat{V}(\phi) \left| B_M^j \right\rangle . \tag{SI-30}$$

Another formulation (suitable for an implementation) can be easily derived taking into account the following considerations:

- A function $f(\phi)$ can be expanded in the basis functions D_M^j employing the projection operator provided in eq. SI-29:

$$|f(\phi)\rangle \approx \hat{O}_v |f(\phi)\rangle = \sum_{j=-M}^{+M} \left[f(\phi_j)\Delta\phi \left| D_M^j(\phi) \right\rangle \right]. \tag{SI-31}$$

  Eq. SI-31 is exact in correspondence of a mesh point, i.e. $\left\langle \phi_{j'} \right| \hat{O}_v \left| f(\phi) \right\rangle = f(\phi_{j'})$.‡‡ If $M \to \infty$ (and therefore $\Delta\phi \to 0$), the last term of eq. SI-31 can be substituted with an integral:

$$\sum_{j=-M}^{+M} \left[f(\phi_j)\Delta\phi \left| D_M^j(\phi) \right\rangle \right] \xrightarrow[M\to\infty]{\Delta\phi\to 0} \int_{-\pi}^{\pi} f(\phi_j)\delta(\phi_j - \phi) d\phi_j, \tag{SI-32}$$

  where $\delta(\phi_j - \phi)$ is the Dirac's function. Eq. SI-31 can be employed to expand a wavefunction (see also eq. 3.11 of ref. SI5):

$$|\psi(\phi)\rangle \approx \sum_{j=-M}^{+M} \left[\psi(\phi_j)\Delta\phi \left| D_M^j(\phi) \right\rangle \right], \tag{SI-33}$$

**The following relationship holds:
$\hat{O}_v^2 = \Sigma_{j'=-M}^{+M} \Sigma_{j=-M}^{+M} \left[\left| D_M^j \right\rangle \Delta\phi \left\langle \phi_j \middle| D_M^{j'} \right\rangle \Delta\phi \left\langle \phi_{j'} \right| \right] = \Sigma_{j'=-M}^{+M} \Sigma_{j=-M}^{+M} \left[\left| D_M^j \right\rangle \Delta\phi D_M^{j'}(\phi_j) \Delta\phi \left\langle \phi_{j'} \right| \right] = \Sigma_{j=-M}^{+M} \left[\left| D_M^j \right\rangle \frac{\Delta\phi}{\Delta\phi} \Delta\phi \left\langle \phi_j \right| \right] = \Sigma_{j=-M}^{+M} \left[\left| D_M^j \right\rangle \Delta\phi \left\langle \phi_j \right| \right] = \hat{O}_v$,
where eq. SI-25 was employed.

††It should be noticed that $\hbar = 1$ in atomic units.

‡‡Because:
$\left\langle \phi_{j'} \right| \hat{O}_v \left| f(\phi) \right\rangle = \Delta\phi \left\langle \phi_{j'} \right| \Sigma_{j=-M}^{+M} \left[f(\phi_j) \left| D_M^j \right\rangle \right] = \Delta\phi f(\phi_{j'}) \left\langle \phi_{j'} \middle| D_M^{j'} \right\rangle = \Delta\phi f(\phi_{j'}) \frac{1}{\Delta\phi} = f(\phi_{j'})$,
where eq. SI-25 was employed.

or to express the first derivative of $D_M^j(\phi)$ (compare eq. SI-34 with eq. SI-15):

$$\frac{dD_M^j}{d\phi} \approx \sum_{j'=-M}^{+M} \left[D'_{j'j} \left| D_M^{j'}(\phi) \right\rangle \right], \tag{SI-34}$$

where eq. SI-28 has been employed.

- An internal product such as the following one:

$$\langle \psi_x \,|\, \psi_y \rangle = \int_{-\pi}^{+\pi} \psi_x^*(\phi) \psi_y(\phi) d\phi, \tag{SI-35}$$

can be approximated as follows:

$$\langle \psi_x \,|\, \psi_y \rangle \approx \langle \psi_x \,|\, \hat{O}_v^\dagger \hat{O}_v \,|\, \psi_y \rangle = \sum_{i=-M}^{+M} \sum_{j=-M}^{+M} \left[\langle \psi_x \,|\, \phi_i \rangle \Delta\phi \left\langle D_M^i \middle| D_M^j \right\rangle \Delta\phi \langle \phi_j \,|\, \psi_y \rangle \right] =$$
$$\sum_{j=-M}^{+M} \left[\langle \psi_x \,|\, \phi_j \rangle \Delta\phi \langle \phi_j \,|\, \psi_y \rangle \right] = \sum_{j=-M}^{+M} \left[\psi_x^*(\phi_j) \psi_y(\phi_j) \Delta\phi \right]; \tag{SI-36}$$

i.e. the employment of the basis functions $D_M^j(\phi)$ allows the evaluation of the integral of eq. SI-35 through discretisation and summation of the integrand values.

- The matrix elements of an operator such as the potential energy operator $\hat{V}(\phi)$ are easily obtained. Employing an orthonormal basis $\left| B_M^j \right\rangle = \sqrt{\Delta\phi} \left| D_M^j \right\rangle$, a single matrix element can be written as follows:

$$\left\langle B_M^{j'} \middle| \hat{V}(\phi) \middle| B_M^j \right\rangle = \Delta\phi \left\langle D_M^{j'} \middle| \hat{V}(\phi) \middle| D_M^j \right\rangle. \tag{SI-37}$$

Eq. SI-37 can be simplified employing the following approximation (see eqs. 4.14 and 4.17 of ref. SI6):

$$\hat{V}(\phi) \left| D_M^j \right\rangle \approx V(\phi_j) \left| D_M^j \right\rangle. \tag{SI-38}$$

Taking into account that $\left\langle D_M^{j'} \middle| D_M^j \right\rangle = \frac{\delta_{j'j}}{\Delta\phi}$, the RHS of eq. SI-37 can be rewritten in the following manner:

$$\Delta\phi \left\langle D_M^{j'} \middle| \hat{V}(\phi) \middle| D_M^j \right\rangle \approx \Delta\phi V(\phi_j) \left\langle D_M^{j'} \middle| D_M^j \right\rangle = \Delta\phi V(\phi_j) \frac{\delta_{j'j}}{\Delta\phi} = V(\phi_j) \delta_{j'j}. \tag{SI-39}$$

The result of eq. SI-39 can be directly employed in the matrix formulation of $\hat{H}^0_{vib}$ (see eq. SI-42).

- The matrix elements of the differential part of the kinetic energy operator* can be derived taking into account eq. SI-34 and applying eq. SI-38 to $g^{\phi\phi}(\phi)$:

$$g^{\phi\phi}(\phi)\left|D^k_M\right\rangle \approx g^{\phi\phi}(\phi_k)\left|D^k_M\right\rangle \tag{SI-40}$$

With these relationships, the differential part of $\hat{H}^0_{vib_{j'j}}$ can be obtained as follows (employing an orthonormal basis $\left|B^j_M\right\rangle = \sqrt{\Delta\phi}\left|D^j_M\right\rangle$):

$$\begin{aligned}
&-\frac{\hbar^2}{2}\left\langle B^{j'}_M\right|\frac{\partial}{\partial\phi}\left[g^{\phi\phi}(\phi)\frac{\partial}{\partial\phi}\right]\left|B^j_M\right\rangle = -\frac{\hbar^2\Delta\phi}{2}\left\langle D^{j'}_M\right|\frac{\partial}{\partial\phi}\left[g^{\phi\phi}(\phi)\frac{\partial}{\partial\phi}\right]\left|D^j_M\right\rangle = \\
&-\frac{\hbar^2\Delta\phi}{2}\left\langle D^{j'}_M\right|\frac{\partial}{\partial\phi}\left\{g^{\phi\phi}(\phi)\sum_{k=-M}^{+M}\left[D'_{kj}\left|D^k_M\right\rangle\right]\right\} = -\frac{\hbar^2\Delta\phi}{2}\left\langle D^{j'}_M\right|\frac{\partial}{\partial\phi}\left\{\sum_{k=-M}^{+M}\left[g^{\phi\phi}(\phi)\left|D^k_M\right\rangle D'_{kj}\right]\right\} \\
&\approx -\frac{\hbar^2\Delta\phi}{2}\left\langle D^{j'}_M\right|\frac{\partial}{\partial\phi}\left\{\sum_{k=-M}^{+M}\left[g^{\phi\phi}(\phi_k)\left|D^k_M\right\rangle D'_{kj}\right]\right\} = -\frac{\hbar^2\Delta\phi}{2}\left\langle D^{j'}_M\right|\frac{\partial}{\partial\phi}\left\{\sum_{k=-M}^{+M}\left[\left|D^k_M\right\rangle g^{\phi\phi}(\phi_k)D'_{kj}\right]\right\} \\
&= -\frac{\hbar^2\Delta\phi}{2}\left\langle D^{j'}_M\right|\left\{\sum_{k=-M}^{+M}\left[\sum_{l=-M}^{+M}\left(D'_{lk}\left|D^l_M\right\rangle\right)g^{\phi\phi}(\phi_k)D'_{kj}\right]\right\} = \\
&-\frac{\hbar^2\Delta\phi}{2}\left\langle D^{j'}_M\right|\left\{\sum_{k=-M}^{+M}\left[\sum_{l=-M}^{+M}\left(\left|D^l_M\right\rangle D'_{lk}\right)g^{\phi\phi}(\phi_k)D'_{kj}\right]\right\} = -\frac{\hbar^2}{2}\sum_{k=-M}^{+M}\left[D'_{j'k}g^{\phi\phi}(\phi_k)D'_{kj}\right];
\end{aligned} \tag{SI-41}$$

where the relationship $\left\langle D^{j'}_M\middle|D^l_M\right\rangle = \frac{\delta_{j'l}}{\Delta\phi}$ has been used. Taking into account eqs. SI-37, SI-39 and SI-41, it is possible to rewrite eq. SI-30 in the following manner:

$$\hat{H}^0_{vib_{j'j}} = -\frac{\hbar^2}{2}\sum_{k=-M}^{+M}\left[D'_{j'k}g^{\phi\phi}(\phi_k)D'_{kj}\right] + \delta_{j'j}V'(\phi_j) + \delta_{j'j}V(\phi_j); \tag{SI-42}$$

where $\delta_{j'j}$ is the Kronecker delta and $g^{\phi\phi}(\phi_k)$, $V'(\phi_j)$ and $V(\phi_j)$ are elements of the diagonal matrices $\mathbf{g}^{\phi\phi}$, $\mathbf{V}'$ and $\mathbf{V}$. $\mathbf{H}^0_{vib}$ is a matrix of dimensions $(2M+1)\times(2M+1)$: the diagonalization of this matrix leads to the desired results (i.e. wavefunctions of $\hat{H}^0_{vib}$ and their energies, which are the eigenvectors and the eigenvalues, respectively, of the eigenvalue problem associated to the matrix $\mathbf{H}^0_{vib}$).

*The pseudopotential term $V'(\phi)$ (see eq. SI-22) is a contribution to the kinetic energy operator, which can be treated analogously to the potential energy term.

2.2.2 Formulation of $\hat{H}^1_{vibrot}$

In the following hamiltonian formulation, rotational motions are explicitly taken into account:

$$\hat{H}_{vibrot} = \overbrace{\frac{1}{2}\left[\hat{p}_\phi g^{\phi\phi}\hat{p}_\phi\right] + \hat{V}' + \hat{V}}^{pseudorotational\ term} + \overbrace{\frac{1}{2}\left[\sum_{\alpha,\beta}^{3}\hat{P}_\alpha g^{\alpha\beta}\hat{P}_\beta\right]}^{rotational\ term} + \overbrace{\frac{1}{2}\left[\sum_{\alpha}^{3}\hat{P}_\alpha(g^{\alpha\phi}\hat{p}_\phi + \hat{p}_\phi g^{\alpha\phi})\right]}^{Coriolis\ term}. \tag{SI-43}$$

In principle, pseudorotational and rotational motions are not separable in eq. SI-43. In practice, the separability of pseudorotational and rotational motions depends on (i) the magnitude of the Coriolis term and (ii) the variation of the values of $g^{\alpha\beta}$ (which are ϕ-dependent and therefore affected by the peudorotational motion).†

In order to provide a matrix formulation of eq. SI-43, a suitable basis must be chosen. Following Meyer,[SI5,SI7] the pseudospectral basis proposed in the previous subsection can be combined with a suitable rotational basis.

Spherical harmonics $Y_J^K(\theta,\varphi)$ are often employed for a quantum mechanical description of the rotational motions of molecular systems. $Y_J^K(\theta,\varphi)$ are eigenfunctions of one of the components of the angular momentum with respect to molecule-fixed axes, while the other two components are involved in more complex relationships.‡ More specifically, the following relationships hold:§

$$\int_0^\pi \left\{d\theta \sin\theta \int_0^{2\pi}\left[d\varphi Y_J^K(\theta,\varphi)^* Y_{J'}^{K'}(\theta,\varphi)\right]\right\} = \left\langle Y_J^K \middle| Y_{J'}^{K'}\right\rangle = \delta_{JJ'}\delta_{KK'}; \tag{SI-44}$$

$$\left\langle Y_{J'}^{K'}\middle|\hat{P}_x\middle|Y_J^K\right\rangle = \frac{\hbar}{2}\delta_{JJ'}\left\{\delta_{K',K-1}\left[\sqrt{J(J+1)-K(K-1)}\right] + \delta_{K',K+1}\left[\sqrt{J(J+1)-K(K+1)}\right]\right\}; \tag{SI-45}$$

$$\left\langle Y_{J'}^{K'}\middle|\hat{P}_y\middle|Y_J^K\right\rangle = -i\frac{\hbar}{2}\delta_{JJ'}\left\{\delta_{K',K-1}\left[\sqrt{J(J+1)-K(K-1)}\right] + \delta_{K',K+1}\left[\sqrt{J(J+1)-K(K+1)}\right]\right\}; \tag{SI-46}$$

$$\left\langle Y_{J'}^{K'}\middle|\hat{P}_z\middle|Y_J^K\right\rangle = \hbar K\delta_{JJ'}\delta_{KK'}; \tag{SI-47}$$

†In other words, the separation of pseudorotational and rotational motions is achieved if the Coriolis term is set equal to zero and the ϕ-dependence of $g^{\alpha\beta}$ is neglected ($g^{\alpha\beta} = const$).

‡and can be used for the construction of ladder (or shift) operators (for example, see table 3.1 of ref. SI8).

§These relationships can be easily found in many articles (see eq. A2.3 of ref. SI5 or eq. 5 of ref. SI9) and textbooks (for example, see refs. SI8 and SI10). A complete treatment of the rotational problem (for example, see chapter 3 of ref. SI8) should include a discussion of the angular momentum components with respect to space-fixed axes, and would require a third quantum number (in addition to J and K).

where J assumes positive integer values ($J = 0, 1, 2, \ldots$) and K can assume integer values in the range $[-J, J]$ (i.e. $J \geq |K|$). In this subsection, only the case $J = 1$ is discussed and therefore K can be equal to -1, 0 and $+1$. The three spherical harmonics Y_1^{-1}, Y_1^0 and Y_1^1 (i.e. the spherical harmonics for the case $J = 1$) can be linearly combined to obtain another basis, which leads to a simpler matrix formulation of $\hat{H}^1_{vibrot}$. More specifically, this approach (suggested and employed by Meyer)[SI5,SI7] leads to a simpler formulation of the matrix elements of $\hat{P}_x$ and $\hat{P}_y$. The new basis is defined by the following transformation:¶

$$\begin{bmatrix} \chi_1 \\ \chi_2 \\ \chi_3 \end{bmatrix} = \frac{1}{\sqrt{2}} \begin{bmatrix} 1 & 0 & -1 \\ -i & 0 & -i \\ 0 & \sqrt{2} & 0 \end{bmatrix} \begin{bmatrix} Y_1^{-1} \\ Y_1^0 \\ Y_1^1 \end{bmatrix}. \tag{SI-48}$$

The orthonormality of the new basis functions can be easily verified employing eqs. SI-44 and SI-48:‖

$$\langle \chi_1 | \chi_1 \rangle = \frac{1}{2} \overbrace{\langle Y_1^{-1} | Y_1^{-1} \rangle}^{1} + \frac{1}{2} \overbrace{\langle Y_1^1 | Y_1^1 \rangle}^{1} - \frac{1}{2} \overbrace{\langle Y_1^{-1} | Y_1^1 \rangle}^{0} - \frac{1}{2} \overbrace{\langle Y_1^1 | Y_1^{-1} \rangle}^{0} = 1; \tag{SI-49}$$

$$\langle \chi_2 | \chi_2 \rangle = -\frac{i^2}{2} \overbrace{\langle Y_1^{-1} | Y_1^{-1} \rangle}^{1} - \frac{i^2}{2} \overbrace{\langle Y_1^1 | Y_1^1 \rangle}^{1} - \frac{i^2}{2} \overbrace{\langle Y_1^{-1} | Y_1^1 \rangle}^{0} - \frac{i^2}{2} \overbrace{\langle Y_1^1 | Y_1^{-1} \rangle}^{0} = -i^2 = 1; \tag{SI-50}$$

$$\langle \chi_3 | \chi_3 \rangle = \langle Y_1^0 | Y_1^0 \rangle = 1; \tag{SI-51}$$

$$\langle \chi_1 | \chi_2 \rangle = -\frac{i}{2} \overbrace{\langle Y_1^{-1} | Y_1^{-1} \rangle}^{1} + \frac{i}{2} \overbrace{\langle Y_1^1 | Y_1^1 \rangle}^{1} - \frac{i}{2} \overbrace{\langle Y_1^{-1} | Y_1^1 \rangle}^{0} + \frac{i}{2} \overbrace{\langle Y_1^1 | Y_1^{-1} \rangle}^{0} = 0; \tag{SI-52}$$

$$\langle \chi_1 | \chi_3 \rangle = \overbrace{\langle Y_1^{-1} | Y_1^0 \rangle}^{0} - \overbrace{\langle Y_1^1 | Y_1^0 \rangle}^{0} = 0; \tag{SI-53}$$

$$\langle \chi_2 | \chi_3 \rangle = i \overbrace{\langle Y_1^{-1} | Y_1^0 \rangle}^{0} + i \overbrace{\langle Y_1^1 | Y_1^0 \rangle}^{0} = 0. \tag{SI-54}$$

Eqs. SI-49 - SI-54 lead to the following relationship:

$$\langle \chi_\alpha | \chi_\beta \rangle = \delta_{\alpha\beta} \quad \text{with} \quad \alpha, \beta = 1, 2, 3; \tag{SI-55}$$

¶The basis functions introduced in eq. SI-48 are often called cubic harmonics.

‖Remember that if $|\chi_2\rangle = -\frac{i}{\sqrt{2}} \left| Y_1^{-1} \right\rangle - \frac{i}{\sqrt{2}} |Y_1^1\rangle$ then $\langle \chi_2 | = \frac{i}{\sqrt{2}} \left\langle Y_1^{-1} \right| + \frac{i}{\sqrt{2}} \langle Y_1^1 |$.

i.e. the new basis functions are orthonormal. The matrix elements of $\hat{P}_x$ can be derived as follows:

$$\langle\chi_1|\hat{P}_x|\chi_1\rangle = \frac{1}{2}\overbrace{\langle Y_1^{-1}|\hat{P}_x|Y_1^{-1}\rangle}^{0\ (see\ eq.\ \text{SI-45})} + \frac{1}{2}\overbrace{\langle Y_1^{1}|\hat{P}_x|Y_1^{1}\rangle}^{0} - \frac{1}{2}\overbrace{\langle Y_1^{-1}|\hat{P}_x|Y_1^{1}\rangle}^{0} - \frac{1}{2}\overbrace{\langle Y_1^{1}|\hat{P}_x|Y_1^{-1}\rangle}^{0} = 0; \tag{SI-56}$$

$$\langle\chi_2|\hat{P}_x|\chi_2\rangle = -\frac{i^2}{2}\overbrace{\langle Y_1^{-1}|\hat{P}_x|Y_1^{-1}\rangle}^{0} - \frac{i^2}{2}\overbrace{\langle Y_1^{1}|\hat{P}_x|Y_1^{1}\rangle}^{0} - \frac{i^2}{2}\overbrace{\langle Y_1^{-1}|\hat{P}_x|Y_1^{1}\rangle}^{0} - \frac{i^2}{2}\overbrace{\langle Y_1^{1}|\hat{P}_x|Y_1^{-1}\rangle}^{0} = 0; \tag{SI-57}$$

$$\langle\chi_3|\hat{P}_x|\chi_3\rangle = \overbrace{\langle Y_1^{0}|\hat{P}_x|Y_1^{0}\rangle}^{0} = 0; \tag{SI-58}$$

$$\langle\chi_1|\hat{P}_x|\chi_2\rangle = -\frac{i}{2}\overbrace{\langle Y_1^{-1}|\hat{P}_x|Y_1^{-1}\rangle}^{0} + \frac{i}{2}\overbrace{\langle Y_1^{1}|\hat{P}_x|Y_1^{1}\rangle}^{0} - \frac{i}{2}\overbrace{\langle Y_1^{-1}|\hat{P}_x|Y_1^{1}\rangle}^{0} + \frac{i}{2}\overbrace{\langle Y_1^{1}|\hat{P}_x|Y_1^{-1}\rangle}^{0} = 0; \tag{SI-59}$$

$$\langle\chi_1|\hat{P}_x|\chi_3\rangle = \frac{1}{\sqrt{2}}\overbrace{\langle Y_1^{-1}|\hat{P}_x|Y_1^{0}\rangle}^{\hbar/\sqrt{2}} - \frac{1}{\sqrt{2}}\overbrace{\langle Y_1^{1}|\hat{P}_x|Y_1^{0}\rangle}^{\hbar/\sqrt{2}} = \frac{\hbar}{2} - \frac{\hbar}{2} = 0; \tag{SI-60}$$

$$\langle\chi_2|\hat{P}_x|\chi_3\rangle = \frac{i}{\sqrt{2}}\overbrace{\langle Y_1^{-1}|\hat{P}_x|Y_1^{0}\rangle}^{\hbar/\sqrt{2}} + \frac{i}{\sqrt{2}}\overbrace{\langle Y_1^{1}|\hat{P}_x|Y_1^{0}\rangle}^{\hbar/\sqrt{2}} = \frac{i\hbar}{2} + \frac{i\hbar}{2} = i\hbar; \tag{SI-61}$$

$$\langle\chi_2|\hat{P}_x|\chi_1\rangle = \langle\chi_1|\hat{P}_x|\chi_2\rangle^* = 0; \tag{SI-62}$$

$$\langle\chi_3|\hat{P}_x|\chi_1\rangle = \langle\chi_1|\hat{P}_x|\chi_3\rangle^* = 0; \tag{SI-63}$$

$$\langle\chi_3|\hat{P}_x|\chi_2\rangle = \langle\chi_2|\hat{P}_x|\chi_3\rangle^* = -i\hbar. \tag{SI-64}$$

Matrix elements of $\hat{P}_y$ and $\hat{P}_z$ can be easily derived in the same manner. If the subscripts x, y and z are labeled, respectively, with the numbers 1, 2 and 3, the 27 matrix elements of $\hat{P}_x$, $\hat{P}_y$ and $\hat{P}_z$ can be readily calculated with the following relationship:

$$\langle\chi_\alpha|\hat{P}_\beta|\chi_\gamma\rangle = -i\hbar\varepsilon_{\alpha\beta\gamma}; \tag{SI-65}$$

where $\varepsilon_{\alpha\beta\gamma}$ is the Levi-Civita symbol.

The basis set employed for the matrix formulation of $\hat{H}_{vibrot}$ in the case $J = 1$ is the following one:[SI5,SI7]

$$\left\{ \left| B_M^j \chi_\alpha \right\rangle \right\} \equiv \left\{ \left| B_M^j \right\rangle \left| \chi_\alpha \right\rangle \right\} \quad \text{with} \quad j = 0, \pm 1, \ldots, \pm M \quad \text{and} \quad \alpha = 1,2,3. \tag{SI-66}$$

The basis set proposed in the previous equation is finite (with a number of elements equal to $3 \times [2M + 1]$) and can be employed in the definition of a projection operator $\hat{O}_{vr}$. In other words, the wavefunction ψ^1_{vibrot} can be represented in terms of the basis set mentioned in SI-66:

$$\psi^1_{vibrot} \approx \hat{O}_{vr} \psi^1_{vibrot} = \sum_{\alpha=1}^{3} \sum_{j=-M}^{+M} \left[c_{j\alpha} \left| B_M^j \chi_\alpha \right\rangle \right]. \tag{SI-67}$$

A partition between pseudorotational and rotational components can be achieved when the following relationship is assumed:

$$c_{j\alpha} = a_j b_\alpha; \tag{SI-68}$$

i.e. when the RHS term of SI-67 can be rewritten as follows:

$$\sum_{\alpha}^{3} \sum_{j=-M}^{+M} c_{j\alpha} \left| B_M^j \chi_\alpha \right\rangle = \sum_{\alpha}^{3} \left[b_\alpha \left(\sum_{j=-M}^{+M} a_j \left| B_M^j \right\rangle \right) \left| \chi_\alpha \right\rangle \right] = \overbrace{\left(\sum_{j=-M}^{+M} a_j \left| B_M^j \right\rangle \right)}^{\psi_{vib}} \overbrace{\left(\sum_{\alpha}^{3} b_\alpha \left| \chi_\alpha \right\rangle \right)}^{\psi_{rot}}. \tag{SI-69}$$

Eq. SI-69 is the starting point of the strategy proposed in this work for the automatic assignment of the eigenstates of $\hat{H}^1_{vibrot}$ to a specific eigenstate of $\hat{H}^0_{vib}$. The n-th eigenstate $\psi^{0(n)}_{vib}$ of the hamiltonian $\hat{H}^0_{vib}$ can be expressed as follows:

$$\psi^{0(n)}_{vib} = \sum_{j=-M}^{+M} a_j^{(n)} \left| B_M^j \right\rangle. \tag{SI-70}$$

In eq. SI-70, the coefficients $a_j^{(n)}$ are a result of the diagonalization of the matrix whose elements ($\hat{H}^0_{vib_{j'j}}$) are provided in eq. SI-42. The representation of the eigenstate $\psi^{0(n)}_{vib}$ can be 'extended' employing the rotational basis set:

$$\psi^{0(n)}_{vib,ext.} = \sum_{\alpha=1}^{3} \sum_{j=-M}^{+M} \left[a_j^{(n)} \left| B_M^j \chi_\alpha \right\rangle \right]. \tag{SI-71}$$

In eq. SI-71, $c_{j1} = c_{j2} = c_{j3} = a_j^{(n)}$ and $b_1 = b_2 = b_3 = 1$ (compare eqs. SI-67 and SI-68 with eq. SI-71). If the partition of pseudorotational and rotational components is assumed, an eigenstate of $\hat{H}^1_{vibrot}$ can be written as follows:

$$\psi^{1(ml)}_{vibrot} = \overbrace{\left(\sum_{j=-M}^{+M} a_j^{(m)} \left| B_M^j \right\rangle \right)}^{\psi^{0(m)}_{vib}} \overbrace{\left(\sum_{\alpha}^{3} b_{\alpha}^{(ml)} \left| \chi_{\alpha} \right\rangle \right)}^{\psi^{1(ml)}_{rot}}, \tag{SI-72}$$

and the internal product between $\psi^{0(n)}_{vib,ext.}$ and $\psi^{1(ml)}_{vibrot}$ can be written as follows (see eq. SI-36):

$$\left\langle \psi^{0(n)}_{vib,ext.} \middle| \psi^{1(ml)}_{vibrot} \right\rangle = \overbrace{\left(\sum_{j=-M}^{+M} a_j^{(n)} a_j^{(m)} \Delta\phi \right)}^{\delta_{nm}} \left(\sum_{\alpha}^{3} b_{\alpha}^{(ml)} \right) = \delta_{nm} \left(\sum_{\alpha}^{3} b_{\alpha}^{(ml)} \right), \tag{SI-73}$$

i.e. if $n \neq m$ then $\left\langle \psi^{0(n)}_{vib,ext.} \middle| \psi^{1(ml)}_{vibrot} \right\rangle = 0$, while (assuming $\sum_{\alpha}^{3} b_{\alpha}^{(ml)} \neq 0$) if $m = n$ then $\left\langle \psi^{0(n)}_{vib,ext.} \middle| \psi^{1(ml)}_{vibrot} \right\rangle \neq 0$. In principle, the partition between pseudorotational and rotational components cannot be assumed when the hamiltonian proposed in eq. SI-43 is employed without simplifying assumptions. In practice, the partition employed in eq. SI-73 is an approximation which explains the usefulness of the following algorithm to carry out the automatic assignment of an eigenstate of $\hat{H}^1_{vibrot}$ to a specific eigenstate of $\hat{H}^0_{vib}$:

1. for a given eigenstate $\psi^{1(x)}_{vibrot}$ of $\hat{H}^1_{vibrot}$, calculate the absolute values of the internal products $\left\langle \psi^{0(n)}_{vib,ext.} \middle| \psi^{1(x)}_{vibrot} \right\rangle$ for all the values of n, which labels the eigenstates of $\hat{H}^0_{vib}$;

2. the eigenstate $\psi^{1(x)}_{vibrot}$ of $\hat{H}^1_{vibrot}$ is assigned to the eigenstate $\psi^{0(n)}_{vib}$ of $\hat{H}^0_{vib}$ corresponding to the value of n for which $\left| \left\langle \psi^{0(n)}_{vib,ext.} \middle| \psi^{1(x)}_{vibrot} \right\rangle \right|$ is maximum.

The matrix formulation of $\hat{H}^1_{vibrot}$ is the following one:

$$\hat{H}^1_{vibrot_{\tau' j', \tau j}} = \left\langle \chi_{\tau'} B_M^{j'} \middle| \left\{ \frac{1}{2} \left[\hat{p}_{\phi} g^{\phi\phi} \hat{p}_{\phi} \right] + \hat{V}' + \hat{V} \right\} \middle| B_M^j \chi_{\tau} \right\rangle + \frac{1}{2} \left\langle \chi_{\tau'} B_M^{j'} \middle| \left[\sum_{\alpha,\beta}^{3} \hat{P}_{\alpha} g^{\alpha\beta} \hat{P}_{\beta} \right] \middle| B_M^j \chi_{\tau} \right\rangle$$
$$+ \frac{1}{2} \left\langle \chi_{\tau'} B_M^{j'} \middle| \left[\sum_{\alpha}^{3} \hat{P}_{\alpha} (g^{\alpha\phi} \hat{p}_{\phi} + \hat{p}_{\phi} g^{\alpha\phi}) \right] \middle| B_M^j \chi_{\tau} \right\rangle . \tag{SI-74}$$

Eq. SI-74 is the starting point for another formulation of $\hat{H}^1_{vibrot}$ which can be easily achieved, if the following considerations are taken into account:

- The first term on the RHS of eq. SI-74 is the pseudorotational contribution, which is discussed in subsection 2.2.1 (see eqs. SI-30 - SI-42): in other words the first term on the RHS of eq. SI-74 is a matrix formulation of $\hat{H}^0_{vib}$, and can be rewritten in the following manner:

$$\left\langle \chi_{\tau'} B^{j'}_M \middle| \left\{ \frac{1}{2}\left[\hat{p}_\phi g^{\phi\phi} \hat{p}_\phi\right] + \hat{V}' + \hat{V} \right\} \middle| B^j_M \chi_\tau \right\rangle =$$
$$\left\langle \chi_{\tau'} \middle| \left\{ -\frac{\hbar^2}{2} \left\langle B^{j'}_M \middle| \frac{\partial}{\partial\phi}\left[g^{\phi\phi}(\phi)\frac{\partial}{\partial\phi}\right] \middle| B^j_M \right\rangle + \left\langle B^{j'}_M \middle| \hat{V}'(\phi) \middle| B^j_M \right\rangle + \left\langle B^{j'}_M \middle| \hat{V}(\phi) \middle| B^j_M \right\rangle \right\} \middle| \chi_\tau \right\rangle =$$
$$\left\langle \chi_{\tau'} \middle| \left\{ -\frac{\hbar^2}{2} \sum_{k=-M}^{+M} \left(D'_{j'k} g^{\phi\phi}(\phi_k) D'_{kj}\right) + \delta_{j'j} V'(\phi_j) + \delta_{j'j} V(\phi_j) \right\} \middle| \chi_\tau \right\rangle =$$
$$-\frac{\hbar^2}{2} \langle \chi_{\tau'} | \chi_\tau \rangle \sum_{k=-M}^{+M} \left(D'_{j'k} g^{\phi\phi}(\phi_k) D'_{kj}\right) + \langle \chi_{\tau'} | \chi_\tau \rangle \delta_{j'j} V'(\phi_j) + \langle \chi_{\tau'} | \chi_\tau \rangle \delta_{j'j} V(\phi_j) =$$
$$-\frac{\hbar^2}{2} \delta_{\tau'\tau} \sum_{k=-M}^{+M} \left(D'_{j'k} g^{\phi\phi}(\phi_k) D'_{kj}\right) + \delta_{\tau'\tau}\delta_{j'j} V'(\phi_j) + \delta_{\tau'\tau}\delta_{j'j} V(\phi_j) = \hat{H}^0_{vib_{\tau'j',\tau j}}. \tag{SI-75}$$

The formulation obtained in eq. SI-75 is equivalent to the one provided in eq. SI-42.**

- The second term on the RHS of eq. SI-74 is the rotational contribution. This contribution is affected by the pseudorotational motion through the ϕ-dependence of $g^{\alpha\beta}$. Taking into account the following approximation (analogous to the ones proposed in eqs. SI-38 and SI-40):

$$g^{\alpha\beta}(\phi) \left| D^k_M \right\rangle \approx g^{\alpha\beta}(\phi_k) \left| D^k_M \right\rangle, \tag{SI-76}$$

and the relationships provided in eqs. SI-55 and SI-65, the rotational contribution can be reformulated as follows:

$$\frac{1}{2}\left\langle \chi_{\tau'} B^{j'}_M \middle| \left[\sum_{\alpha,\beta}^{3} \hat{P}_\alpha g^{\alpha\beta}(\phi) \hat{P}_\beta\right] \middle| B^j_M \chi_\tau \right\rangle = \frac{1}{2}\left\langle \chi_{\tau'} B^{j'}_M \middle| \left\{\sum_{\alpha,\beta}^{3} \hat{P}_\alpha \left[g^{\alpha\beta}(\phi) \left| B^j_M \right\rangle\right] \hat{P}_\beta\right\} \middle| \chi_\tau \right\rangle$$
$$\approx \frac{1}{2}\left\langle \chi_{\tau'} B^{j'}_M \middle| \left\{\sum_{\alpha,\beta}^{3} \hat{P}_\alpha \left[g^{\alpha\beta}(\phi_j) \left| B^j_M \right\rangle\right] \hat{P}_\beta\right\} \middle| \chi_\tau \right\rangle = \frac{1}{2}\left\langle B^{j'}_M \middle| B^j_M \right\rangle \left\langle \chi_{\tau'} \middle| \left\{\sum_{\alpha,\beta}^{3} \hat{P}_\alpha g^{\alpha\beta}(\phi_j) \hat{P}_\beta\right\} \middle| \chi_\tau \right\rangle$$
$$= -\frac{i\hbar}{2}\delta_{j'j}\left\langle \chi_{\tau'} \middle| \left\{\sum_{\alpha,\beta,\gamma}^{3} \hat{P}_\alpha g^{\alpha\beta}(\phi_j)\varepsilon_{\gamma\beta\tau} \middle| \chi_\gamma \right\rangle\right\} = -\frac{i\hbar}{2}\delta_{j'j}\left\langle \chi_{\tau'} \middle| \left\{\sum_{\alpha,\beta,\gamma}^{3} \hat{P}_\alpha \middle| \chi_\gamma \right\rangle g^{\alpha\beta}(\phi_j)\varepsilon_{\gamma\beta\tau}\right\}$$

**There is a difference: the presence of the factors $\delta_{\tau'\tau}$ in eq. SI-75, which is due to the employment of a larger basis set.

$$
\begin{aligned}
&= \frac{i^2\hbar^2}{2}\delta_{j'j}\Big\langle \chi_{\tau'} \Big| \Big\{ \sum_{\alpha,\beta,\gamma,\zeta}^{3} \varepsilon_{\zeta\alpha\gamma} \Big| \chi_\zeta \Big\rangle g^{\alpha\beta}(\phi_j)\varepsilon_{\gamma\beta\tau} \Big\} = -\frac{\hbar^2}{2}\delta_{j'j}\langle\chi_{\tau'}\,|\,\chi_\zeta\rangle \sum_{\alpha,\beta,\gamma,\zeta}^{3} \Big[\varepsilon_{\zeta\alpha\gamma} g^{\alpha\beta}(\phi_j)\varepsilon_{\gamma\beta\tau}\Big] \\
&= -\frac{\hbar^2}{2}\delta_{j'j}\delta_{\tau'\zeta} \sum_{\alpha,\beta,\gamma,\zeta}^{3} \Big[\varepsilon_{\zeta\alpha\gamma} g^{\alpha\beta}(\phi_j)\varepsilon_{\gamma\beta\tau}\Big] = -\frac{\hbar^2}{2}\delta_{j'j} \sum_{\alpha,\beta,\gamma}^{3} \Big[\varepsilon_{\tau'\alpha\gamma} g^{\alpha\beta}(\phi_j)\varepsilon_{\gamma\beta\tau}\Big].
\end{aligned}
\tag{SI-77}
$$

- The Coriolis term (third term on the RHS of eq. SI-74) accounts for the interactions between pseudorotational and rotational motions through (i) the ϕ-dependence of the six matrix elements $g^{\alpha\phi}$ and (ii) contributions which involve $\hat{P}_\alpha$ and $\hat{p}_\phi$. If the same approximation proposed in eqs. SI-38, SI-40 and SI-76 (for $\hat{V}(\phi)$, $g^{\phi\phi}(\phi)$ and $g^{\alpha\beta}(\phi)$, respectively) is adopted for $g^{\alpha\phi}$ the Coriolis term can be rewritten as follows (eqs. SI-34, SI-55 and SI-65 are employed):

$$
\begin{aligned}
&\frac{1}{2}\Big\langle \chi_{\tau'}B_M^{j'} \Big| \Big[\sum_\alpha^3 \hat{P}_\alpha(g^{\alpha\phi}\hat{p}_\phi + \hat{p}_\phi g^{\alpha\phi})\Big] \Big| B_M^{j}\chi_\tau \Big\rangle = \\
&-\frac{i\hbar\Delta\phi}{2}\Big\langle \chi_{\tau'}D_M^{j'} \Big| \Big[\sum_\alpha^3 \hat{P}_\alpha g^{\alpha\phi}(\phi)\frac{\partial}{\partial\phi}\Big] \Big| D_M^{j}\chi_\tau \Big\rangle - \frac{i\hbar\Delta\phi}{2}\Big\langle \chi_{\tau'}D_M^{j'} \Big| \Big[\sum_\alpha^3 \hat{P}_\alpha \frac{\partial}{\partial\phi} g^{\alpha\phi}(\phi)\Big] \Big| D_M^{j}\chi_\tau \Big\rangle = \\
&-\frac{i\hbar\Delta\phi}{2}\Big\langle \chi_{\tau'}D_M^{j'} \Big| \sum_\alpha^3 \Big\{\hat{P}_\alpha g^{\alpha\phi}(\phi) \sum_{k=-M}^{+M}\Big[D'_{kj}\,\big|D_M^k\big\rangle\Big]\Big\} \Big| \chi_\tau \Big\rangle - \frac{i\hbar\Delta\phi}{2}\Big\langle \chi_{\tau'}D_M^{j'} \Big| \sum_\alpha^3 \Big\{\hat{P}_\alpha \frac{\partial}{\partial\phi} g^{\alpha\phi}(\phi)\,\big|D_M^{j}\big\rangle\Big\} \Big| \chi_\tau \Big\rangle \approx \\
&-\frac{i\hbar\Delta\phi}{2}\Big\langle \chi_{\tau'}D_M^{j'} \Big| \sum_\alpha^3 \Big\{\hat{P}_\alpha \sum_{k=-M}^{+M}\Big[\big|D_M^k\big\rangle g^{\alpha\phi}(\phi_k)D'_{kj}\Big]\Big\} \Big| \chi_\tau \Big\rangle - \frac{i\hbar\Delta\phi}{2}\Big\langle \chi_{\tau'}D_M^{j'} \Big| \sum_\alpha^3 \Big\{\hat{P}_\alpha \frac{\partial}{\partial\phi}\big|D_M^{j}\big\rangle g^{\alpha\phi}(\phi_j)\Big\} \Big| \chi_\tau \Big\rangle = \\
&-\frac{i\hbar\Delta\phi}{2}\Big\langle \chi_{\tau'} \Big| \sum_\alpha^3 \Big\{\hat{P}_\alpha \sum_{k=-M}^{+M}\Big[\Big\langle D_M^{j'}\Big| D_M^k\Big\rangle g^{\alpha\phi}(\phi_k)D'_{kj}\Big]\Big\} \Big| \chi_\tau \Big\rangle - \frac{i\hbar\Delta\phi}{2}\Big\langle \chi_{\tau'}D_M^{j'} \Big| \sum_\alpha^3 \Big\{\hat{P}_\alpha \sum_{k=-M}^{+M}\Big[D'_{kj}\,\big|D_M^k\big\rangle\Big] g^{\alpha\phi}(\phi_j)\Big\} \Big| \chi_\tau \Big\rangle = \\
&-\frac{i\hbar\Delta\phi}{2}\Big\langle \chi_{\tau'} \Big| \sum_\alpha^3 \Big\{\hat{P}_\alpha \sum_{k=-M}^{+M}\Big[\frac{\delta_{j'k}}{\Delta\phi} g^{\alpha\phi}(\phi_k)D'_{kj}\Big]\Big\} \Big| \chi_\tau \Big\rangle - \frac{i\hbar\Delta\phi}{2}\Big\langle \chi_{\tau'} \Big| \sum_\alpha^3 \Big\{\hat{P}_\alpha \sum_{k=-M}^{+M}\Big[\Big\langle D_M^{j'}\Big| D_M^k\Big\rangle D'_{kj}\Big] g^{\alpha\phi}(\phi_j)\Big\} \Big| \chi_\tau \Big\rangle = \\
&-\frac{i\hbar}{2}\Big\langle \chi_{\tau'} \Big| \sum_\alpha^3 \Big\{\hat{P}_\alpha g^{\alpha\phi}(\phi_{j'})D'_{j'j}\Big\} \Big| \chi_\tau \Big\rangle - \frac{i\hbar\Delta\phi}{2}\Big\langle \chi_{\tau'} \Big| \sum_\alpha^3 \Big\{\hat{P}_\alpha \sum_{k=-M}^{+M}\Big[\frac{\delta_{j'k}}{\Delta\phi} D'_{kj}\Big] g^{\alpha\phi}(\phi_j)\Big\} \Big| \chi_\tau \Big\rangle = \\
&-\frac{i\hbar}{2}\Big\langle \chi_{\tau'} \Big| \sum_\alpha^3 \Big\{\hat{P}_\alpha \Big| \chi_\tau \Big\rangle g^{\alpha\phi}(\phi_{j'})D'_{j'j}\Big\} - \frac{i\hbar}{2}\Big\langle \chi_{\tau'} \Big| \sum_\alpha^3 \Big\{\hat{P}_\alpha D'_{j'j} g^{\alpha\phi}(\phi_j)\Big\} \Big| \chi_\tau \Big\rangle = \\
&\frac{i^2\hbar^2}{2}\Big\langle \chi_{\tau'} \Big| \sum_{\alpha,\beta}^3 \Big\{\varepsilon_{\beta\alpha\tau} \Big| \chi_\beta \Big\rangle g^{\alpha\phi}(\phi_{j'})D'_{j'j}\Big\} + \frac{i^2\hbar^2}{2}\Big\langle \chi_{\tau'} \Big| \sum_{\alpha,\beta}^3 \Big\{\varepsilon_{\beta\alpha\tau} \Big| \chi_\beta \Big\rangle D'_{j'j} g^{\alpha\phi}(\phi_j)\Big\} = \\
&-\frac{\hbar^2}{2}\Big\{\sum_{\alpha,\beta}^3 \Big[\delta_{\tau'\beta}\varepsilon_{\beta\alpha\tau}g^{\alpha\phi}(\phi_{j'})D'_{j'j}\Big] + \sum_{\alpha,\beta}^3 \Big[\delta_{\tau'\beta}\varepsilon_{\beta\alpha\tau}D'_{j'j}g^{\alpha\phi}(\phi_j)\Big]\Big\} = -\frac{\hbar^2}{2}\Big\{\sum_{\alpha}^3 \Big[\varepsilon_{\tau'\alpha\tau}g^{\alpha\phi}(\phi_{j'})D'_{j'j}\Big] + \sum_{\alpha}^3 \Big[\varepsilon_{\tau'\alpha\tau}D'_{j'j}g^{\alpha\phi}(\phi_j)\Big]\Big\}.
\end{aligned}
\tag{SI-78}
$$

Eqs. SI-75, SI-77 and SI-78 can be used to rewrite eq. SI-74 in the following manner:[SI5]

$$\hat{H}^{1}_{vibrot_{\tau' j',\tau j}} = -\frac{\hbar^2}{2}\left\{\delta_{\tau'\tau}\sum_{k=-M}^{+M}\left[D'_{j'k}g^{\phi\phi}(\phi_k)D'_{kj}\right] + \delta_{j'j}\sum_{\alpha,\beta,\gamma}^{3}\left[\varepsilon_{\tau'\alpha\gamma}g^{\alpha\beta}(\phi_j)\varepsilon_{\gamma\beta\tau}\right] + \sum_{\alpha}^{3}\left[\varepsilon_{\tau'\alpha\tau}g^{\alpha\phi}(\phi_{j'})D'_{j'j}\right] + \sum_{\alpha}^{3}\left[\varepsilon_{\tau'\alpha\tau}D'_{j'j}g^{\alpha\phi}(\phi_j)\right]\right\} + \delta_{\tau'\tau}\delta_{j'j}\left[V'(\phi_j) + V(\phi_j)\right]. \quad \text{(SI-79)}$$

$\mathbf{H}^{1}_{vibrot}$ is a matrix of dimensions $3(2M+1)\times 3(2M+1)$: the formulation provided in eq. SI-79 was implemented and employed for the calculation of $\psi^{1(x)}_{vibrot}$ and $E^{1(x)}_{vibrot}$ (eigenvectors and eigenvalues of $\mathbf{H}^{1}_{vibrot}$, respectively).[††] In other words, the quantities of interest are obtained from the solutions of the eigenvalue problem associated to $\mathbf{H}^{1}_{vibrot}$.

[††]In the main text, the eigenvalues are labeled $E_v[1_{01}]$, $E_v[1_{11}]$ and $E_v[1_{10}]$: the subscript v is referred to the eigenstate of $\mathbf{H}^{0}_{vib}$ to which the eigenstates of $\mathbf{H}^{1}_{vibrot}$ are assigned.

3 The 1D-NSE as a Sturm-Liouville problem

In the main text of the article, solutions to the 1D-NSE are provided. These solutions were computed by means of a suitable numerical method (see the previous section). An analysis of the various contributions to the final solution can be performed starting with a simple analytical treatment. The following discussion is limited to the case $J = 0$.

From the mathematical point of view, the 1D-NSE along the pseudorotation angle ϕ (equation 8 in the main text of the article) is a Sturm-Liouville problem with periodic boundary conditions. The problem can be formulated as follows (in atomic units $\hbar = 1$):

$$L\psi(\phi) = -\frac{d}{d\phi}\left[\frac{1}{2}g^{\phi\phi}(\phi)\frac{d\psi(\phi)}{d\phi}\right] + P(\phi)\psi(\phi) = \lambda\psi(\phi); \tag{SI-80}$$

$$\psi(0) = \psi(2\pi); \tag{SI-81}$$

$$\frac{d\psi(0)}{d\phi} = \frac{d\psi(2\pi)}{d\phi}; \tag{SI-82}$$

where $P(\phi)$ (eq. SI-80) is equal to the sum of potential (V) and pseudopotential (V') terms and the periodic boundary conditions are specified in eqs. SI-81 and SI-82.

The solutions of the problem specified with eqs. SI-80 - SI-82 (i.e. λ and $\psi(\phi)$) are affected by $g^{\phi\phi}(\phi)$ and $P(\phi)$.

3.1 First case: $g^{\phi\phi}(\phi) = const$ and $P(\phi) = const$

When $g^{\phi\phi}(\phi) = const$ and $P(\phi) = const$ exact analytical solutions to the Sturm-Liouville problem are available. If the values $g^{\phi\phi}(\phi) = 2$ and $P(\phi) = 0$ are employed, eq. SI-80 can be written as follows:

$$-\frac{d^2\psi(\phi)}{d\phi^2} = \lambda\psi(\phi). \tag{SI-83}$$

Solutions of eq. SI-83 can be easily derived employing the boundary conditions specified in eqs. SI-81 and SI-82. More specifically, if $\lambda > 0$ the following differential equation is obtained (where $\lambda = \omega^2$):

$$\psi'' + \omega^2\psi = 0. \tag{SI-84}$$

The general solution of eq. SI-84 can be written in the following manner:

$$\psi(\phi) = c_1\cos(\omega\phi) + c_2\sin(\omega\phi); \tag{SI-85}$$

where c_1 and c_2 are constants. In order to find the positive eigenvalues of eq. SI-84, the general solution provided in eq. SI-85 is employed in eqs. SI-81 and SI-82:

$$\overbrace{c_1}^{\psi(0)} = \overbrace{c_1\cos(2\pi\omega)+c_2\sin(2\pi\omega)}^{\psi(2\pi)}; \tag{SI-86}$$

$$\overbrace{c_2}^{\psi'(0)} = \overbrace{c_2\cos(2\pi\omega)-c_1\sin(2\pi\omega)}^{\psi'(2\pi)}. \tag{SI-87}$$

If $c_1 \neq 0$ and $c_2 \neq 0$, eqs. SI-86 and SI-87 are satisfied when:

$$\cos(2\pi\omega)=1 \qquad \text{and} \qquad \sin(2\pi\omega)=0; \tag{SI-88}$$

i.e. when:

$$\omega = n \implies \lambda = n^2 \quad \text{with} \quad n=1,2,3,\ldots. \tag{SI-89}$$

There are two linearly independent eigenfuctions corresponding to the same eigenvalue $\lambda = n^2$:*

$$\cos(n\phi) \qquad \text{and} \qquad \sin(n\phi). \tag{SI-90}$$

If $\lambda = 0$, the general solution of the differential equation SI-84 is the following one:

$$\psi(\phi) = c_1 + c_2\phi; \tag{SI-91}$$

which is simplified to $\psi(\phi) = c_1$ when the periodic boundary condition $\psi(0) = \psi(2\pi)$ is employed. Therefore, to the solutions provided in eqs. SI-89 and SI-90 must be added another solution with eigenvalue $\lambda = 0$, corresponding to an eigenfunction equal to a constant (conventionally, the number 1 is chosen). There are no solutions to eq. SI-83 with $\lambda < 0$.

Eigenvalues and eigenfunctions of eq. SI-83 are shown in fig. SI 2a.†

With the same procedure previously outlined, analytical solutions can be obtained also in the case of $P(\phi) = const \neq 0$: in this case, eq. SI-83 can be rewritten in the following manner:

$$-\frac{d^2\psi(\phi)}{d\phi^2} + K\psi(\phi) = \lambda\psi(\phi) \implies -\frac{d^2\psi(\phi)}{d\phi^2} = (\lambda - K)\psi(\phi); \tag{SI-92}$$

*More generally, the two linearly independent eigenfunctions are $\cos(n\phi+\varphi)$ and $\sin(n\phi+\varphi)$, where the value of φ must be the same if the two eigenfunctions correspond to the same eigenvalue n^2; otherwise, φ can assume different values.

†Eigenfunctions provided in SI-90 are not normalized. When the normalization is not taken into account, eigenfunctions are specified up to a numerical constant. Even when the normalization is imposed (and the numerical constant specified), the eigenfuction ψ_n and its negative counterpart $-\psi_n$ are exactly the same solution, i.e. ψ_n is specified up to a sign.

where K is a numerical constant.[‡] Eigenfunctions of eq. SI-92 are exactly the same of eq. SI-83 (given in SI-90), but the eigenvalues are shifted according to the value of K:

$$\lambda - K = n^2 \implies \lambda = K + n^2 \quad \text{with} \quad n = 0, 1, 2, 3, \ldots . \tag{SI-93}$$

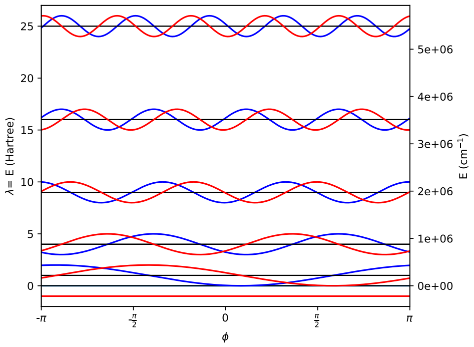

(a) $\frac{1}{2}g^{\phi\phi}(\phi) = 1; P(\phi) = 0.$

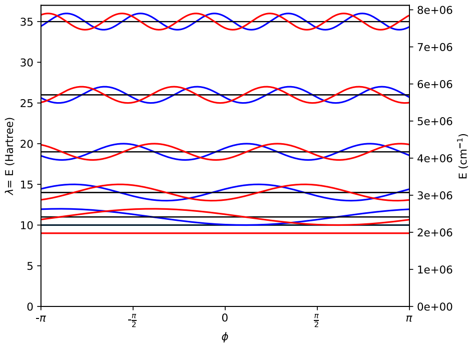

(b) $\frac{1}{2}g^{\phi\phi}(\phi) = 1; P(\phi) = 10$ (Hr).

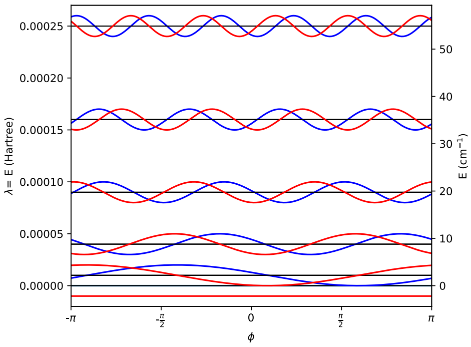

(c) $\frac{1}{2}g^{\phi\phi}(\phi) = 1 \cdot 10^{-5}; P(\phi) = 0.$

Figure SI 2: Solutions of the Sturm-Liouville problem when $g^{\phi\phi}(\phi) = const$ and $P(\phi) = const$. The black horizontal lines in the three graphs are the eigenvalues. Energy scale is provided in Hartree (i.e. atomic units) on the left side of the graph and in cm^{-1} on the right side. The eigenfunctions are not normalized.

Eigenvalues and eigenfunctions of eq. SI-92 are plotted in fig. SI 2b. On the right of figs. SI 2a and SI 2b, the energy is provided in terms of cm^{-1}: it is clear that the order of magnitude of the eigenvalues is not realistic for the systems investigated in this work. The reason is the choice of the $g^{\phi\phi}(\phi)$ value; therefore, a reformulation of eq. SI-83 with $g^{\phi\phi}(\phi) = const \neq 2$ can lead to more realistic values (with the choice of a value of $g^{\phi\phi}(\phi) \ll 1$):

[‡]Eq. SI-92 is written in atomic units, therefore K is expressed in Hartree (Hr).

$$-X\frac{d^2\psi(\phi)}{d\phi^2} = \lambda\psi(\phi) \implies -\frac{d^2\psi(\phi)}{d\phi^2} = \left(\frac{\lambda}{X}\right)\psi(\phi). \tag{SI-94}$$

The eigenfunctions of eq. SI-94 are the the same of eqs. SI-83 and SI-92, while the eigenvalues are scaled according to the value of X:

$$\frac{\lambda}{X} = n^2 \implies \lambda = Xn^2 \quad \text{with} \quad n = 0,1,2,3,\ldots. \tag{SI-95}$$

In fig. SI 2c eigenvalues and eigenfunctions of eq. SI-94 are provided: transition energies (i.e. the energy difference between two eigenvalues) are lower than the ones observed in figs. SI 2a and SI 2b. The results reported in this subsection are far from the results reported in the main article for 1,2-dioxolane, 1,2-oxathiolane and 3-chloro-1,2-dithiolane. However, the patterns reported in fig. SI 2 can be considered a rough approximation of the results expected for a molecular system with extremely low potential energy barriers to the pseudorotation. For example, cyclopentane was described as a free pseudorotor in refs. SI11 and SI12.

3.2 Second case: $g^{\phi\phi}(\phi) = const$ and $P(\phi) = f(\phi)$

When $g^{\phi\phi}(\phi) = const$ and $P(\phi) = V(\phi) + V'(\phi) \neq const$ (where $V(\phi)$ and $V'(\phi)$ are the potential and the psedopotential of one of the systems investigated in this work, respectively), the Sturm-Liouville problem is more complex. However, the increased complexity of the mathematical problem corresponds to a better description of the molecular systems under investigation. With $g^{\phi\phi}(\phi) = const$, eq. SI-80 can be written as follows:

$$-\frac{1}{2}g^{\phi\phi}\frac{d^2\psi(\phi)}{d\phi^2} + P(\phi)\psi(\phi) = \lambda\psi(\phi). \tag{SI-96}$$

A comparison between the solutions of eq. SI-96 and the solutions of the more general problem (when $g^{\phi\phi}(\phi) = f(\phi)$) allows an estimation of the quantitative importance of the structural relaxation effects. These effects are due to the dependence of $g^{\phi\phi}(\phi)$ and $V'(\phi)$ from the pseudorotation angle ϕ:[§] as a consequence, their relevance can be assessed neglecting the dependence of $g^{\phi\phi}$ and V' (but retaining the dependence of V) from ϕ.

The availability of an exact analytical solution of the Sturm-Liouville problem proposed in eq. SI-96 (with the boundary conditions specified in eqs. SI-81 and SI-82) depends on the functional form of $P(\phi) = V(\phi) + V'(\phi)$. However, a systematic procedure to obtain approximated analytical solutions to eq. SI-96 is well known: this procedure is named WKB approximation and is detailed in many textbooks of mathematical methods (for example, see chapter 10 of ref. SI13). A discussion of the WKB approximation is beyond the scope of this work: the procedure is mentioned in order to underline the existence of another route to the solution

[§] In order to perform the calculation of $g^{\phi\phi}$ and V' at a specific value of ϕ, the elements of the classical kinetic energy matrix are needed (see eq. SI-11).

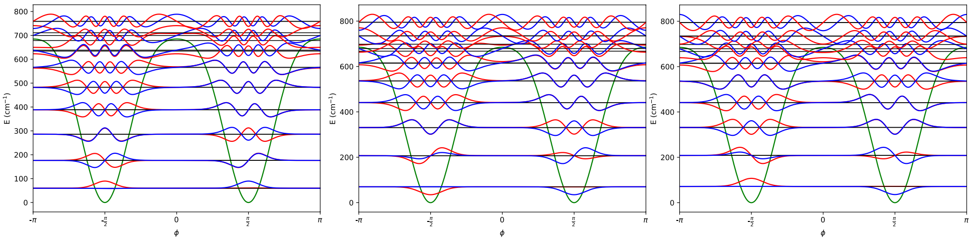

(a) $g^{\phi\phi}(\phi) = f(\phi) \neq const$; $P(\phi) = V(\phi) + V'(\phi)$.

(b) $g^{\phi\phi}(\phi) \approx 3.83 \cdot 10^{-5}$; $P(\phi) = V(\phi) + V'(\phi)$.

(c) $g^{\phi\phi}(\phi) \approx 3.83 \cdot 10^{-5}$; $P(\phi) = V(\phi)$.

Figure SI 3: Sturm-Liouville problem associated to the pseudorotation of the 1,2-dioxolane molecule: graphical representation of numerical solutions (the first 19 eigenstates are shown). The continuous green line is the potential $V(\phi)$. Subfigure SI 3a is reported in the main text of the article (see subfigure 4a).

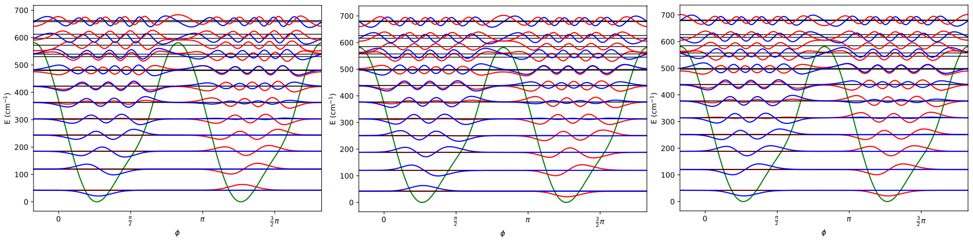

(a) $g^{\phi\phi}(\phi) = f(\phi) \neq const$; $P(\phi) = V(\phi) + V'(\phi)$.

(b) $g^{\phi\phi}(\phi) \approx 2.91 \cdot 10^{-5}$; $P(\phi) = V(\phi) + V'(\phi)$.

(c) $g^{\phi\phi}(\phi) \approx 2.91 \cdot 10^{-5}$; $P(\phi) = V(\phi)$.

Figure SI 4: Sturm-Liouville problem associated to the pseudorotation of the 1,2-oxathiolane molecule: graphical representation of numerical solutions (the first 23 eigenstates are shown). The continuous green line is the potential $V(\phi)$. Subfigure SI 4a is reported in the main text of the article (see subfigure 4b).

of eq. SI-96, which is alternative to the numerical method proposed and employed in this work.

With the method proposed in this work (see the previous section of this document and the main text of the article), numerical solutions to eq. SI-96 can be easily obtained. In this manner, the effects of the ϕ-dependencies of $g^{\phi\phi}$, V and V' on the solutions of the 1D-NSE are assessed. More specifically, the 1D-NSE is solved (i) taking into account the ϕ-dependencies of all the contributions ($g^{\phi\phi}$, V and V'), (ii) taking into account the ϕ-dependencies of V and V' but neglecting the ϕ-dependence of $g^{\phi\phi}$ (in this case $g^{\phi\phi}(\phi) = const$, where *const* is a constant value equal to the arithmetic mean of the $g^{\phi\phi}$ values calculated at each angle ϕ) and (iii) taking into account the ϕ-dependence of V and neglecting the ϕ-dependencies of $g^{\phi\phi}$ and V' (in this case, $V'(\phi) = 0$). The results (shown in figures SI 3 - SI 5 and summarised in tables SI 1 - SI 3) lead to the following considerations:¶

¶the discussion proposed in this section concerns the case $J = 0$ only. Moreover, the following

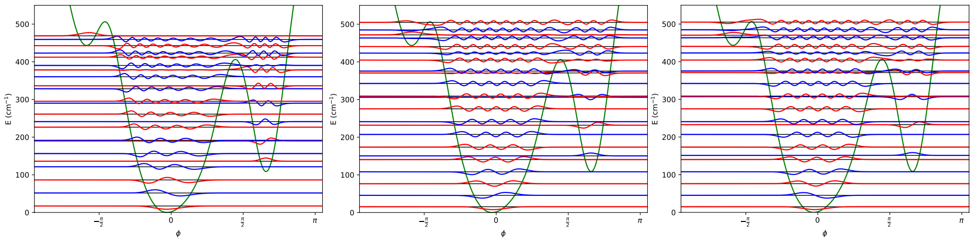

(a) $g^{\phi\phi}(\phi) = f(\phi) \neq const$; $P(\phi) = V(\phi) + V'(\phi)$.
(b) $g^{\phi\phi}(\phi) \approx 9.17 \cdot 10^{-6}$; $P(\phi) = V(\phi) + V'(\phi)$.
(c) $g^{\phi\phi}(\phi) \approx 9.17 \cdot 10^{-6}$; $P(\phi) = V(\phi)$.

Figure SI 5: Sturm-Liouville problem associated to the pseudorotation of the 3-chloro-1,2-dithiolane molecule: graphical representation of numerical solutions (the first 23 eigenstates are shown). The continuous green line is the potential $V(\phi)$. The results shown in subfigure SI 5a are reported in the main text of the article (see subfigure 4c).

- The contribution of $V'(\phi)$ to the solutions of the 1D-NSE is extremely small.[‖] This consideration is suggested by a comparison between the values listed in the third and in the fourth columns of tables SI 1, SI 2 and SI 3 and confirmed by a comparison between subfigures SI 3b and SI 3c (in the case of 1,2-dioxolane), subfigures SI 4b and SI 4c (with regards to 1,2-oxathiolane) and subfigures SI 5b and SI 5c (3-chloro-1,2-dithiolane): the eigenvalues listed in the third and in the fourth columns of the tables are almost equal, with only small differences (never larger than 2 cm^{-1}, often smaller than 1 cm^{-1}); the differences in the graphical representations referred to the same molecule are exclusively due to differences of sign (ψ_v and $-\psi_v$ are representations of the same eigenstate).

- The contribution due to the ϕ-dependence of $g^{\phi\phi}$ to the solutions of the 1D-NSE is not negligible. The effects of the ϕ-dependence of $g^{\phi\phi}$ on the eigenvalues of the 1D-NSE are particularly evident in the case of 1,2-dioxolane (see the numerical values listed in the second and in the third column of table SI 1). With regards to 1,2-oxathiolane the effects of the ϕ-dependence of $g^{\phi\phi}$ is less pronounced than in the other two cases (compare second and third column of table SI 2 and subfigures SI 4a and SI 4b). The number of pseudorotational eigenstates localised above the second minimum (inside the second well) of the 1D-PES of 3-chloro-1,2-dithiolane is affected by the ϕ-dependence of $g^{\phi\phi}$: in subfigure SI 5a there are six eigenstates prevalently localised inside the second well of the 1D-PES, while in subfigure SI 5b (which shows the solutions obtained when $g^{\phi\phi}(\phi) = const$) there are only

considerations should be extended with caution to other molecular systems (i.e. molecular systems different from the three discussed in this work).

[‖]With regards to the ring-puckering motion in small molecular systems, this fact was already recognised by Laane and coworkers.[SI14]

four eigenstates prevalently localized inside the second well of the 1D-PES. These data suggest that a careful account of the ϕ-dependence of $g^{\phi\phi}$ is important for an accurate formulation of the 1D-NSE.

- As can be expected, the role of the potential energy term $V(\phi)$ is pivotal. The results shown in a single figure (referred to a specific molecular systems with different choices about $g^{\phi\phi}$ and V') are qualitatively similar: this is because the potential energy term is exactly the same in the three subfigures (see figures SI 3 - SI 5). When the eigenstates with higher values of v are examined, a pattern similar to the one see in figure SI 2 emerge, i.e. the pattern of figure SI 2 is observed only for eigenvalues higher than the top of the potential energy barriers. A description of the solutions of the 1D-NSE at energy higher than the potential energy barriers is of little physical interest in this work.

4 Choice of the reference system: the case of 1,2-dioxolane

A reference system must be chosen to specify the molecular structures and to formulate the kinetic energy operators of $\hat{H}^0_{vib}$ and $\hat{H}^1_{vibrot}$. For the characterisation of internal rotations or pseudorotational motions, PAS is widely adopted as reference system (see for example chapter 9 of ref. SI8). As specified at the end of section 3 in the main text, the results proposed in section 4 of the main text were calculated adopting the PAS as reference system. An alternative route can be chosen: one of the axis of the reference system can be oriented in a special direction, in order to reduce the coupling terms between (overall) rotation and pseudorotational motions. On the other hand, principal axes and reference axes do not coincide in this reference system. A comparison between the two approaches can be found in ref. SI15.

The choice proposed in our contribution (PAS adopted as reference system) is motivated by the features of the molecular systems investigated: in these systems the pseudorotational motion involves the entire molecular structure, and there is not a simple definition of a suitable rotation axis for the (internal) pseudorotational motion. In the case of 1,2-dioxolane, the adoption of the PAS as reference system leads to a deviation from the expected patterns of the values of $\Delta E_v[1_{01}]$, $\Delta E_v[1_{11}]$ and C_v as functions of the index v (see figure 9c and table 1 of the main text). To verify the origin of these deviations, in the case of 1,2-dioxolane the nuclear problem was formulated and solved employing another reference system, defined as follows: the z axis of the cartesian framework of the original Cremer-Pople formulation is retained, while the x axis lies on the Cremer-Pople mean plane (which is perpendicular to the z axis), oriented according to the projection (on the Cremer-Pople mean plane) of the position of the atom labeled with 1 (see figure 2 of the main text) and the y axis is oriented perpendicularly to the xz plane. The origin of the cartesian framework coincides with the center of mass of the entire molecule.

v	E (cm^{-1})		
	figure SI 3a	figure SI 3b	figure SI 3c
0	59.84	69.84	71.55
1	59.84	69.84	71.55
2	176.42	206.98	208.33
3	176.42	206.98	208.33
4	286.15	330.80	331.67
5	286.15	330.80	331.67
6	388.36	440.89	441.26
7	388.36	440.89	441.26
8	482.24	536.33	536.32
9	482.26	536.39	536.39
10	565.74	614.52	614.11
11	566.07	615.78	615.40
12	634.07	667.40	666.85
13	638.02	679.11	678.65
14	678.42	698.06	697.53
15	699.47	734.59	734.16
16	710.91	735.55	735.14
17	758.59	794.52	794.13
18	758.86	795.04	794.72

Table SI 1: 1,2-dioxolane molecule. Eigenvalues associated to the eigenstates shown in figure SI 3. The numerical values reported in the second column are listed in the article (see the second column of table 1).

v	E (cm^{-1})		
	figure SI 4a	figure SI 4b	figure SI 4c
0	42.23	42.23	41.95
1	42.23	42.23	41.95
2	119.53	120.33	120.17
3	119.53	120.33	120.17
4	184.78	188.01	188.27
5	184.78	188.01	188.27
6	243.78	250.56	251.07
7	243.79	250.57	251.08
8	303.01	313.56	313.92
9	303.03	313.59	313.95
10	363.08	376.84	377.09
11	363.17	376.96	377.21
12	422.68	438.58	438.71
13	423.1	439.14	439.28
14	479.74	496.13	496.13
15	481.89	499.14	499.20
16	530.46	545.07	544.86
17	539.47	557.34	557.34
18	572.1	584.73	584.28
19	597.24	616.06	615.92
20	612.23	626.41	626.06
21	658.03	678.46	678.36
22	662.52	681.04	680.78

Table SI 2: 1,2-oxathiolane molecule. Eigenvalues associated to the eigenstates shown in figure SI 4. The numerical values reported in the second column are listed in the article (see the second column of table 2).

v	E (cm^{-1})		
	figure SI 5a	figure SI 5b	figure SI 5c
0	17.15	15.46	15.04
1	51.65	45.65	45.30
2	86.35	76.50	76.22
3	121.26	108.13	107.86
4	136.08	140.58	140.28
5	156.38	150.33	151.24
6	189.79	173.68	173.33
7	191.53	207.30	206.92
8	226.51	231.91	232.92
9	241.2	241.23	240.84
10	261.14	275.29	274.89
11	290.13	305.99	307.10
12	295.19	309.21	308.82
13	328.35	342.62	342.24
14	336.25	370.61	371.20
15	360.23	375.60	375.44
16	378.5	404.76	404.41
17	390.32	423.29	423.26
18	412.21	440.66	440.66
19	423.11	463.55	463.50
20	442.87	471.73	470.22
21	459.34	484.87	484.89
22	468.93	504.84	505.14

Table SI 3: 3-chloro-1,2-dithiolane molecule. Eigenvalues associated to the eigenstates shown in figure SI 5. The numerical values reported in the second column are listed in the article (see the second column of table 3).

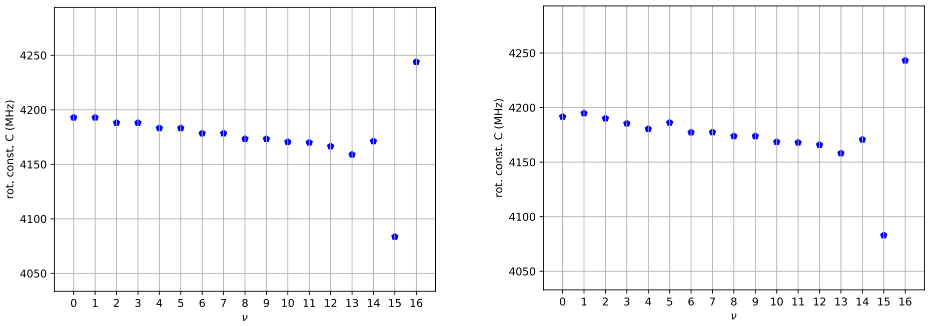

(a) ZAS reference system

(b) PAS reference system

Figure SI 6: Rotational constant C of 1,2-dioxolane as function of the index ν.

This reference configuration is named zeta axis system (ZAS) because the "special" direction of this coordinate system coincides with the zeta axis (which is the same used to specify Cremer-Pople coordinates). A comparison of the results obtained with the PAS and the ZAS reference systems is presented in table SI 4 and fig. SI 6: when the ZAS reference system is employed, the expected patterns of $\Delta E_\nu[1_{01}]$, $\Delta E_\nu[1_{11}]$ and C_ν (with respect to the index ν) are found. In the case of 1,2-dioxolane, the center of mass of the entire molecule and the geometrical center of the 5 atoms directly involved in the central ring are close. As a consequence, in this case the ZAS is close to the cartesian framework employed for the definition of the Cremer-Pople coordinates, and leads to lower values of the coupling terms between rotational and pseudorotational motions without the shortcoming of a dramatic increase of the elements $g_{\alpha\beta}$ (with $\alpha \neq \beta$). In the cases of 1,2-oxathiolane and 3-chloro-1,2-dithiolane this strategy is not suitable, and only the PAS reference system was adopted for the formulation of the nuclear problem.

v	ZAS							PAS						
	E_v (cm^{-1})	$\Delta E_v[1_{01}]$ (MHz)	$\Delta E_v[1_{11}]$ (MHz)	$\Delta E_v[1_{10}]$ (MHz)	A_v (MHz)	B_v (MHz)	C_v (MHz)	E_v (cm^{-1})	$\Delta E_v[1_{01}]$ (MHz)	$\Delta E_v[1_{11}]$ (MHz)	$\Delta E_v[1_{10}]$ (MHz)	A_v (MHz)	B_v (MHz)	C_v (MHz)
0	59.84	11530.2	11711	14855.3	7518	7337.3	4192.9	59.84	11529.3	11709.5	14855.5	7517.9	7337.6	4191.6
1	59.84	11530.2	11711	14855.3	7518	7337.3	4192.9	59.84	11532.4	11712.7	14855.5	7517.9	7337.6	4194.8
2	176.42	11518.7	11709.7	14852.2	7521.6	7330.6	4188.1	176.42	11521.7	11711.3	14852.8	7521.2	7331.6	4190.1
3	176.42	11518.7	11709.7	14852.2	7521.6	7330.6	4188.1	176.42	11517.1	11706.7	14852.8	7521.2	7331.6	4185.5
4	286.15	11507.4	11708.2	14849.1	7525	7324.2	4183.2	286.15	11506.3	11704.7	14850.1	7524.3	7325.9	4180.4
5	286.15	11507.4	11708.2	14849.1	7525	7324.2	4183.2	286.15	11512.0	11710.5	14850.1	7524.3	7325.8	4186.2
6	388.36	11496.5	11706.4	14846.1	7528	7318	4178.4	388.36	11497.5	11704.4	14847.4	7527.2	7320.3	4177.3
7	388.36	11496.4	11706.4	14846	7528	7318	4178.4	388.36	11497.7	11704.6	14847.4	7527.2	7320.2	4177.4
8	482.24	11485.4	11704.6	14843.3	7531.3	7312.1	4173.4	482.24	11488.4	11704.0	14844.8	7530.2	7314.6	4173.8
9	482.26	11484.7	11704.6	14842.6	7531.2	7311.3	4173.3	482.26	11487.9	11704.0	14844.3	7530.2	7314.1	4173.8
10	565.74	11474.3	11703.4	14836.6	7532.8	7303.8	4170.5	565.74	11475.2	11700.8	14838.9	7532.2	7306.7	4168.6
11	566.07	11475.5	11702.6	14838.2	7532.6	7305.6	4170	566.07	11476.2	11700.0	14840.2	7532.0	7308.2	4168.0
12	634.07	11460.6	11706.5	14834	7540	7294	4166.6	634.07	11462.2	11704.9	14835.5	7539.1	7296.4	4165.8
13	638.03	11456.5	11696.5	14835.1	7537.6	7297.5	4159	638.02	11458.2	11694.9	14836.8	7536.7	7300.1	4158.2
14	678.42	11452.7	11720.3	14830.5	7549.1	7281.5	4171.2	678.42	11453.6	11719.1	14831.3	7548.4	7282.9	4170.7
15	699.47	11375.5	11625	14833.3	7541.4	7291.9	4083.6	699.47	11376.8	11623.8	14834.7	7540.8	7293.9	4082.9
16	710.91	11524.6	11794.2	14830.8	7550.2	7280.6	4244	710.91	11524.6	11793.2	14831.5	7550.1	7281.4	4243.2

Table SI 4: Eigenvalues of $\hat{H}^0_{vib}$ (E_v), lowest rotational transition energies ($J = 0 \rightarrow J = 1$) and rotational constants (A_v, B_v and C_v) associated to the pseudorotational motion of 1,2-dioxolane: comparison between the numerical values calculated with ZAS and PAS reference systems.

References

[SI1] D. Cremer and J. A. Pople. General definition of ring puckering coordinates. *Journal of the American Chemical Society*, 97(6):1354–1358, 1975.

[SI2] D. Cremer. Calculation of puckered rings with analytical gradients. *The Journal of Physical Chemistry*, 94(14):5502–5509, 1990.

[SI3] L. Paoloni, S. Rampino, and V. Barone. Potential-energy surfaces for ring-puckering motions of flexible cyclic molecules through cremer-pople coordinates: Computation, analysis, and fitting. *Journal of Chemical Theory and Computation*, 15(7):4280–4294, 2019.

[SI4] M. J. Frisch, G. W. Trucks, H. B. Schlegel, G. E. Scuseria, M. A. Robb, J. R. Cheeseman, G. Scalmani, V. Barone, G. A. Petersson, H. Nakatsuji, X. Li, M. Caricato, A. V. Marenich, J. Bloino, B. G. Janesko, R. Gomperts, B. Mennucci, H. P. Hratchian, J. V. Ortiz, A. F. Izmaylov, J. L. Sonnenberg, D. Williams-Young, F. Ding, F. Lipparini, F. Egidi, J. Goings, B. Peng, A. Petrone, T. Henderson, D. Ranasinghe, V. G. Zakrzewski, J. Gao, N. Rega, G. Zheng, W. Liang, M. Hada, M. Ehara, K. Toyota, R. Fukuda, J. Hasegawa, M. Ishida, T. Nakajima, Y. Honda, O. Kitao, H. Nakai, T. Vreven, K. Throssell, J. A. Montgomery, Jr., J. E. Peralta, F. Ogliaro, M. J. Bearpark, J. J. Heyd, E. N. Brothers, K. N. Kudin, V. N. Staroverov, T. A. Keith, R. Kobayashi, J. Normand, K. Raghavachari, A. P. Rendell, J. C. Burant, S. S. Iyengar, J. Tomasi, M. Cossi, J. M. Millam, M. Klene, C. Adamo, R. Cammi, J. W. Ochterski, R. L. Martin, K. Morokuma, O. Farkas, J. B. Foresman, and D. J. Fox. Gaussian 16 Revision A.03, 2016. Gaussian Inc. Wallingford CT.

[SI5] R. Meyer. Flexible models for intramolecular motion, a versatile treatment and its application to glyoxal. *Journal of Molecular Spectroscopy*, 76(1):266 – 300, 1979.

[SI6] R. Meyer. Trigonometric interpolation method for one-dimensional quantum-mechanical problems. *The Journal of Chemical Physics*, 52(4):2053–2059, 1970.

[SI7] R. Meyer and E. Bright Wilson. Rotational constants of torsionally excited molecules. *The Journal of Chemical Physics*, 53(10):3969–3980, 1970.

[SI8] H.W. Kroto. *Molecular Rotation Spectra*. Dover books on physics and chemistry. Dover Publications, 1992.

[SI9] Gilbert W. King, R. M. Hainer, and Paul C. Cross. The asymmetric rotor i. calculation and symmetry classification of energy levels. *The Journal of Chemical Physics*, 11(1):27–42, 1943.

[SI10] D.A. McQuarrie and J.D. Simon. *Physical Chemistry. A Molecular Approach.* University Science Books, 1997.

[SI11] John E. Kilpatrick, Kenneth S. Pitzer, and Ralph Spitzer. The thermodynamics and molecular structure of cyclopentane1. *Journal of the American Chemical Society*, 69(10):2483–2488, 1947.

[SI12] J. R. Durig and D. W. Wertz. Vibrational spectra and structure of small-ring compounds. x. spectroscopic evidence for pseudorotation in cyclopentane. *The Journal of Chemical Physics*, 49(5):2118–2121, 1968.

[SI13] C.M. Bender and S.A. Orszag. *Advanced Mathematical Methods for Scientists and Engineers I. Asymptotic Methods and Perturbation Theory*. Springer-Verlag, 1999.

[SI14] Matthew A. Harthcock and Jaan Laane. Evaluation of pseudopotential terms for the vibrational hamiltonian of small ring molecules. *Journal of Molecular Spectroscopy*, 94(2):461 – 462, 1982.

[SI15] Chun C. Lin and Jerome D. Swalen. Internal rotation and microwave spectroscopy. *Rev. Mod. Phys.*, 31:841–892, Oct 1959.
